# Phosphorylated CtIP bridges DNA to promote annealing of broken ends

**DOI:** 10.1073/pnas.2008645117

**Published:** 2020-08-19

**Authors:** Robin Öz, Sean M. Howard, Rajhans Sharma, Hanna Törnkvist, Ilaria Ceppi, Sriram KK, Erik Kristiansson, Petr Cejka, Fredrik Westerlund

**Affiliations:** ^a^Department of Biology and Biological Engineering, Chalmers University of Technology, SE 41296 Gothenburg, Sweden;; ^b^Institute for Research in Biomedicine, Universitá della Svizzera italiana, CH 6500 Bellinzona, Switzerland;; ^c^Department of Biology, Institute of Biochemistry, Eidgenössische Technische Hochschule Zürich, CH 8093 Zurich, Switzerland;; ^d^Department of Mathematical Sciences, Chalmers University of Technology, SE 41296 Gothenburg, Sweden

**Keywords:** CtIP, nanofluidics, single DNA molecule biophysics, homologous recombination, DNA repair

## Abstract

The early steps of DNA double-strand break (DSB) repair in human cells involve the MRE11-RAD50-NBS1 (MRN) complex and its cofactor, phosphorylated CtIP. The roles of these proteins in nucleolytic DSB resection are well characterized, but their role in bridging the DNA ends for efficient and correct repair is much less explored. Here we study the binding of phosphorylated CtIP, which promotes the endonuclease activity of MRN, to single long (∼50 kb) DNA molecules using nanofluidic channels and compare it to the yeast homolog Sae2. CtIP bridges DNA in a manner that depends on the oligomeric state of the protein, and truncated mutants demonstrate that the bridging depends on CtIP regions distinct from those that stimulate the nuclease activity of MRN. Sae2 is a much smaller protein than CtIP, and its bridging is significantly less efficient. Our results demonstrate that the nuclease cofactor and structural functions of CtIP may depend on the same protein population, which may be crucial for CtIP functions in both homologous recombination and microhomology-mediated end-joining.

## Significance

The DNA of our cells is constantly exposed to various types of damaging agents. One of the most critical types of damage is when both strands of the DNA break, and thus such breaks need to be efficiently repaired. It is known that CtIP promotes nucleases in DNA break repair. Here we show that CtIP can also hold the two DNA strands together in solution when DNA is free to move, using novel methodology that allows the monitoring of thousands of single DNA molecules in nanofabricated devices. DNA bridging likely facilitates the enzymatic repair steps and identifies novel CtIP functions that are crucial for repairing broken DNA.

Although estimated to occur only 10 times per day per cell, DNA double-strand breaks (DSBs) represent one of the most toxic forms of DNA damage (1). Failure in repairing DSBs may lead to chromosome mis-segregation and cellular lethality, while incorrect repair may result in mutagenesis and cancer. DSBs can be repaired by either of two main mechanisms: homologous recombination (HR) or end-joining, which includes canonical nonhomologous end-joining (NHEJ) and microhomology-mediated end-joining (MMEJ) (2, 3). All these pathways have been studied extensively using cellular and biochemical assays; however, most of the assays performed to date fail to provide useful information on how the DSB repair proteins help stabilize the broken DNA molecules in 3D space and with respect to one another.

Single-molecule techniques have revolutionized our understanding of DNA–protein interactions by providing details that are not readily accessible with traditional bulk methods (4), including, for example, the stochasticity of reactions, structural changes in DNA, and forces generated as the interactions occur. Nanofluidics has evolved as a powerful tool for studying single DNA molecules and their static and dynamic interactions with different analytes, such as DNA intercalators (5) and proteins (6–10). The method is based on confining DNA in nanofluidic channels with dimensions in the 100-nm regime, forcing the DNA to stretch and accommodate within the spatial confinement (11, 12). The nanoconfined DNA is suspended in solution, leaving the ends free and unobstructed. This means that it is possible to characterize reactions occurring on DNA ends, and also that two long DNA molecules can be positioned in close proximity to allow them to interact and to observe these interactions in real time (7, 13). Thus, nanofluidics is highly suitable for studies of DSB repair.

HR is typically initiated by the resection of the 5′-terminated DNA strand near the DSB, forming a 3′-terminated single-stranded DNA (ssDNA) overhang. DSB resection in human cells is initiated by the MRE11-RAD50-NBS1 (MRN) complex together with phosphorylated CtIP. Upon phosphorylation, the primary known function of CtIP is to promote the endonucleolytic activity of the MRN complex, which preferentially cleaves the 5′-terminated DNA past protein blocks (14–16). The MRN complex, together with CtIP, competes with the main NHEJ factor DNA-PK. Recently, Deshpande et al. (17) demonstrated, on the single DNA molecule level using DNA curtains, how CtIP and MRN can remove DNA-PK from DNA ends through endonucleolytic incision and hence channel repair from NHEJ to HR. Beyond HR, CtIP and MRN are also important for MMEJ, which mediates the joining of two DNA molecules with short microhomologies at the ends (18). The underlying mechanisms of MRN and CtIP in MMEJ are not clear but likely involve both catalytic and structural functions. In contrast, canonical NHEJ is CtIP-independent, while MRN likely plays a supportive role (3, 19–21). Beyond MRN, phosphorylated CtIP also promotes the DNA2-BLM/WRN nuclease-helicase complex that functions downstream of MRN (22, 23). In *Saccharomyces cerevisiae*, the CtIP homolog is Sae2. Just as CtIP, phosphorylated Sae2 promotes the endonuclease activity of the MRN homolog MRX (24).

While the mechanisms of nucleolytic resection by MRN and CtIP are becoming clearer (16, 17, 25), there is an important, nuclease-independent function of both MRN and CtIP in bridging and stabilizing DNA ends (26–28). Although DNA bridging is most likely critical to facilitate the catalytic functions in DSB repair by both end-joining and recombination, it remains poorly understood. The DSB repair components involved, the mechanism by which the DNA bridging occurs, and the consequence of disrupted bridging functions for the respective DSB repair pathways, remain to be clarified. To this point, the MRN complex has been proposed to bridge DNA molecules, which was based mainly on atomic force microscopy (AFM) experiments in which two MR complexes, connected by the zinc hook at the apex of the coiled coils of RAD50, were observed to link two linear DNA molecules (28). However, it has also been observed that the Zn hook may link two MRN complexes that are adjacent to each other on the same DNA molecule (29); thus, the involvement and requirement for MRN in DNA bridging is not fully understood. More recently, the CtIP homolog in *Schizosaccharomyces pombe,* Ctp1, was observed to form filaments along double-stranded DNA (dsDNA) that promote DNA repair through DNA bridging, independent of additional factors (26). This closely agrees with the findings reported in a study by Wilkinson et al. (27) in which human CtIP was shown to anneal short DNA constructs with various complex ends. Both studies were based on AFM imaging, which gives information only on the static DNA–protein interactions for DNA deposited on a surface, leaving important features of the interaction between DNA and CtIP in solution unassessed, such as the conformational changes of genomic length DNA on CtIP binding, the transient bridging ability of the protein, potential sequence specificity of CtIP, as well as the dynamics of the DNA–CtIP interactions. Furthermore, CtIP phosphorylation, which is required for its function to promote MRN (16), was found to be inhibitory for DNA binding and annealing (27), raising questions as to whether the same population of CtIP can carry out both functions.

To better understand the structural role of CtIP in the repair of DSBs, we here establish a nanofluidic assay that allows us to monitor static and dynamic interactions between DNA and phosphorylated CtIP on the single DNA molecule level. Using lambda-phage DNA (λ-DNA) with complementary single-stranded overhangs (12 nt long) as a model DNA, we report that CtIP promotes intramolecular self-annealing of the overhangs by bridging DNA. A library of mutants reveals the regions of the protein that are crucial for efficient bridging. Based on our results, crucial hallmarks in the early stage of DSB repair can potentially be explained, such as a possible role of CtIP in keeping the broken DNA ends in close proximity to enable simultaneous DNA end resection of both broken ends or promoting DNA annealing in MMEJ. We believe that our findings will contribute to a more detailed understanding of the structural function of CtIP in DSB repair.

## Results

### Nanofluidics Allows Detection and Differentiation between Linear and Circular DNA.

**Fig. 1.**
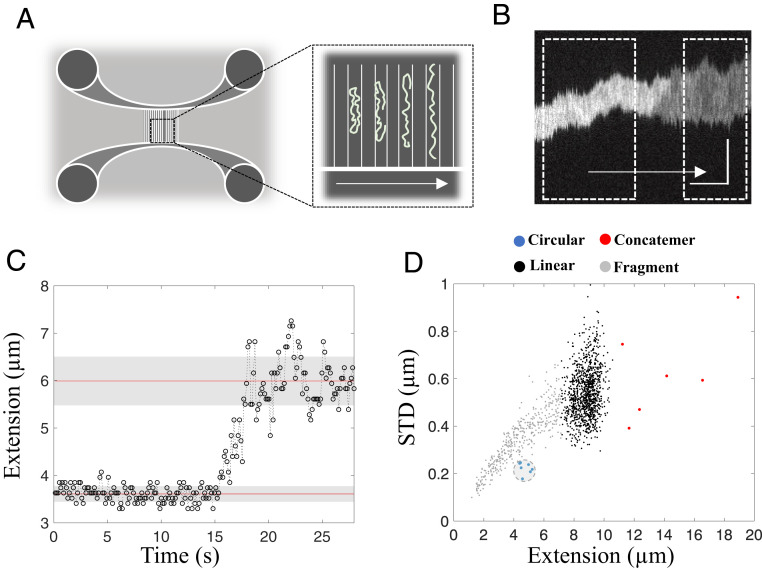
Linearization of a confined circular DNA molecule in a nanofluidic channel. (*A*) Schematic illustration of a nanofluidic device with four loading reservoirs, two horizontal microchannels, and multiple vertical nanochannels. The magnification of the nanochannels visualizes the spontaneous unfolding of a schematic circular DNA molecule with time (in the direction of the arrow). (*B*) Representative kymograph showing the characteristic unfolding of a confined circular λ-DNA–CtIP complex, where the boxed regions highlight the circular (*Left*) and linear (*Right*) conformations of the molecule with time elapsing in the direction of the arrow. The vertical and horizontal scale bars correspond to 5 µm and 5 s, respectively. (*C*) The change in DNA extension with time upon unfolding of DNA from the circular to the linear conformation. The circular conformation has a mean extension of 3.6 µm (bottom red line) with an STD of 0.2 µm (bottom gray patch). The linear conformation has a mean extension of 6.0 µm (top red line) with a corresponding STD of 0.5 µm (top gray patch). (*D*) Scatterplot of molecule extension vs. STD for a control sample of λ-DNA (*n* = 1,512). The circular fraction is shown in blue (marked by a gray circle), full-size linear λ-DNA molecules are in black, concatemers are in red, and linear DNA fragments are in gray.

The 48.5-kbp-long dsDNA of the lambda-phage (λ-DNA) has complementary 12-nt ssDNA overhangs at each end. λ-DNA can attain a circular conformation by annealing of its overhangs and can also form concatemers, in which multiple molecules are joined via intermolecular hybridization of the complementary ends (7). Using nanofluidic channels (schematically shown in Fig. 1*A*), with dimensions of 150 × 100 nm^2^ (details in *Methods*), we are able to characterize and distinguish these different DNA structures. Circular DNA can be readily distinguished from linear DNA molecules in nanofluidic channels by two means (Fig. 1 *B* and *C*) (30, 31). First, circular DNA has approximately twice the amount of DNA per channel segment, which leads to double the fluorescence emission intensity from the intercalated YOYO-1 dye used to visualize the DNA. Second, the polymer physics of DNA change due to the higher local DNA density, which results in significantly lower thermal fluctuations along the DNA extension for a circular DNA molecule (30). The difference in thermal fluctuations can be quantified by the standard deviation (STD) of the extension, calculated from the variation in molecule extension over time (Fig. 1*C*). Photodamage during microscopy imaging can convert circular DNA molecules into their linear conformation in situ (32) via the formation of DSBs within the nanochannels (Fig. 1*B*). On breaking, the double-folded state is no longer thermodynamically favorable, and the DNA will unfold to its linear conformation (30).

Fig. 1*D* shows the extension vs. STD plotted for λ-DNA. Two main DNA species are found: intact linear λ-DNA molecules with an average extension of 8.8 ± 0.6 µm (black) and fragmented linear λ-DNA molecules with shorter extensions (gray). As expected, there is a linear dependence between the extension and STD for linear DNA. In addition, a very small fraction of molecules (<1%) are observed (blue), with lower STD and approximately half the extension of intact full-length λ-DNA (black), which we consider to be circular λ-DNA molecules (30, 31). Furthermore, a few molecules (<1%) with extensions larger than full-size λ-DNA were observed (red), hereinafter referred to as concatemers (7). In this study, circularization of λ-DNA by hybridization of the single-stranded overhangs was used as a reporter of DNA bridging by the CtIP protein.

### CtIP Promotes Circularization of λ-DNA.

To promote the nuclease activity of MRN, CtIP needs to be phosphorylated (16). Although CtIP was previously shown to bind DNA by various methods, this binding was strongly inhibited by CtIP phosphorylation (16, 27). This paradox raised the question of whether stimulation of MRN and DNA binding are mutually exclusive activities, mediated by two different subpopulations of CtIP. Assessing the interaction of phosphorylated CtIP with DNA using a traditional electrophoretic mobility shift assay did not reveal conclusive data on the binding ability of the protein to neither linear nor circular DNA substrates (*SI Appendix*, Fig. S1). To address this matter, we performed single DNA molecule analysis using nanofluidics to detect robust DNA binding of CtIP in its active, phosphorylated form in solution (*SI Appendix*, Fig. S2). Importantly, this preparation of CtIP is capable of promoting the endonuclease activity of MRN (*SI Appendix*, Fig. S3), as reported previously (16).

**Fig. 2.**
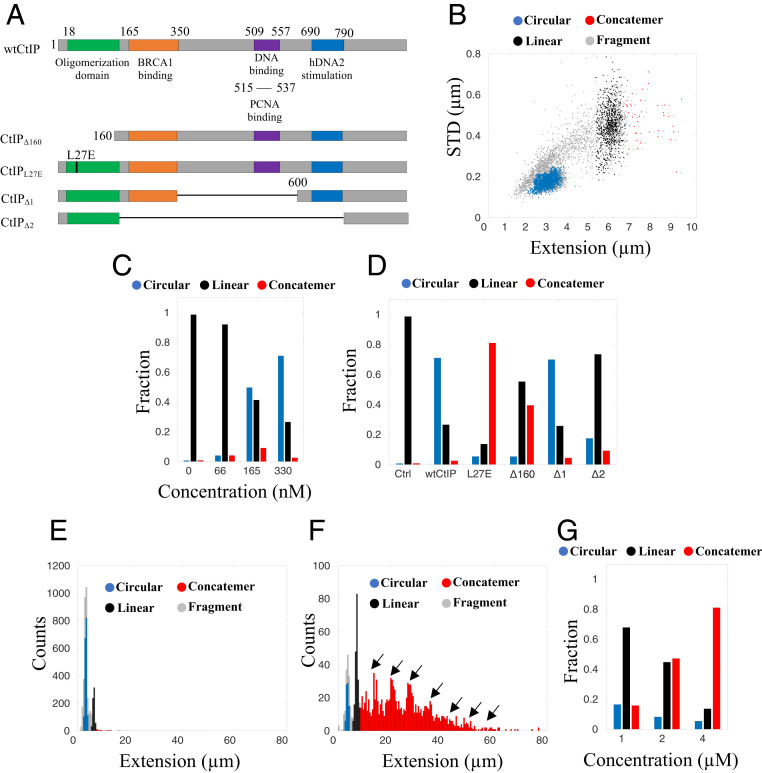
The tetrameric structure of CtIP is important for circularization of λ-DNA. (*A*) Schematic illustration of the CtIP derivatives used in this study, with color-coded regions highlighting different domains of interest. The numbers correspond to the position in the protein sequence. (*B*) Scatterplot of molecule extension vs. STD for λ-DNA (4 µM bp) incubated with wtCtIP (330 nM), equivalent to 500 tetramers per DNA end (*n* = 3,886). Clustering of the datasets was performed to distinguish the circularized λ-DNA molecules (blue) from the full-size linear λ-DNA molecules (black), concatemers (red), and linear fragments (gray). (*C* and *D*) Relative fractions of circular and linear complexes and concatemers at a DNA concentration of 4 µM bp for different concentrations of wtCtIP (*C*) and different derivatives of the protein at a constant protein concentration of 330 nM (*D*). (*E* and *F*) Size histograms for wtCtIP (*n* = 3,886; bin size = 0.5 µm) (*E*) and CtIP_L27E_ (*n* = 1,541; bin size = 0.5 µm) (*F*) at 330 nM protein and 4 µM bp DNA (concentration equivalent to 500 tetramers or 1,000 dimers per DNA end, respectively). The high frequency of concatemers for CtIP_L27E_ compared with wtCtIP is denoted by arrows in the histogram. (*G*) Relative fractions of circular and linear complexes and concatemers at different concentrations of λ-DNA in the presence of CtIP_L27E_ at a constant DNA:protein ratio corresponding to 1,000 dimers per DNA end. Increasing the DNA concentration promotes concatemer formation.

To characterize the interactions between DNA and CtIP, they were mixed in bulk to allow complex formation. Before the solution was loaded onto the nanofluidic chip, sodium dodecyl sulphate was added at a concentration of 0.05% to reduce the nonspecific sticking of the DNA-protein complexes to the channel walls (*Methods*). We analyzed the binding of five CtIP variants to λ-DNA (Fig. 2*A*); the wild-type protein (wtCtIP) and the mutants CtIP_L27E_, CtIP_Δ160_, CtIP_Δ1_, and CtIP_Δ2_ (22, 33). wtCtIP has previously been reported to attain a tetrameric structure in its active state for DNA damage repair, where the N-terminal domain mediates the interaction that generates a head-to-head association between preformed CtIP dimers, to form a dumbbell-shaped structure (15, 27). This dimer–dimer interaction is blocked by replacing the leucine residue at position 27 by glutamic acid (CtIP_L27E_), allowing CtIP dimers as the highest order of tertiary structure (15). The deletion of 160 residues from the N terminus entirely disrupts the oligomeric structure of CtIP due to the absence of the zipper-like domain (45 to 160) (34), likely resulting in a monomeric protein (CtIP_Δ160_) (35). CtIP_Δ1_ lacks residues 350 to 600, including a known DNA-binding motif, while CtIP_Δ2_ lacks residues 165 to 790, corresponding to the entire region between the tetramerization domain and the C-terminal domain, which yields a protein similar to the *S. cerevisiae* CtIP homolog Sae2 (22). In this study, all CtIP derivatives were evaluated in their multiphosphorylated state (*SI Appendix*, Fig. S2), as was done in previous biochemical studies (16, 36).

Fig. 2*B* shows a scatterplot of extension vs. STD as 330 nM wtCtIP (monomer concentration) was added to 4 µM (bp) λ-DNA, equivalent to 500 tetramers per DNA end (∼50 bp per tetramer; details in *Methods*). The plot differs from that of λ-DNA alone (Fig. 1*D*) in several important ways. The most striking observation is the formation of a large cluster of molecules centered at an extension around 3 µm with a low STD (blue), suggesting that they are circular DNA molecules. To quantify the fraction of circles, we used a hierarchical clustering method (details in *Methods*). The addition of wtCtIP increased the circularization of the DNA significantly, yielding a circularization efficiency (*E*_c_) of 71.0% while also generating ∼2.5% concatemers. The DNA circularization depended strongly on the CtIP concentration. A reduced ratio of wtCtIP per λ-DNA end yielded fewer circular DNA complexes. At 66 nM wtCtIP (100 tetramers per end), *E*_c_ was as low as 4.0%, while 165 nM wtCtIP (250 tetramers per DNA end) yielded an *E*_c_ of 49.7% (Fig. 2*C*). Importantly, the circularization and concatemerization efficiencies are used as readouts in our in vitro assay to understand the behavior of the DNA-bound protein, rather than reflecting possible biological implications in vivo, where circularization likely will not be relevant.

To elucidate whether the increased circularization of λ-DNA in the presence of CtIP is dependent on the tetrameric nature of the protein, CtIP_L27E_ was added to DNA. At 330 nM CtIP_L27E_ (1,000 CtIP_L27E_ dimers per λ-DNA end), *E*_c_ was 5.4%, significantly lower than for wtCtIP. The *E*_c_ was found to be 5.3% for 2,000 monomeric CtIP_Δ160_ per λ-DNA end (Fig. 2*D*). This strongly implies that the tetrameric structure plays an important role in self-annealing of the complementary sticky ends of λ-DNA. Interestingly, from the size distribution histograms (Fig. 2 *E* and *F*), it is clear that a large fraction of the CtIP_L27E_–λ-DNA complexes were concatemers (∼81% of all molecules, fragments excluded) with complexes of up to eight hybridized λ-DNA molecules formed. This suggests that CtIP_L27E_ can promote annealing of the complementary ends of λ-DNA. The low number of circles compared to concatemers shows that intermolecular annealing is more likely to occur when the bridging ability of CtIP is impaired, as for CtIP_L27E_. This finding supports the existence of a competition between circularization and concatemer formation, with the dimeric nature of CtIP_L27E_ limiting the intramolecular bridging of DNA but still promoting the intermolecular annealing of DNA ends.

To further explore the capability of CtIP_L27E_ to form concatemers, we performed experiments at different DNA concentrations but the same DNA:protein ratios. While formation of concatemers is an intermolecular process that depends on the total DNA concentration, circularization is an intramolecular process that is concentration-independent (7). The number of concatemers decreased significantly with decreasing total DNA concentration (Fig. 2*G*), in agreement with our hypothesis, while the fraction of circles increased. Concatemers were also formed in the presence of monomeric CtIP_Δ160_ (∼40%; *SI Appendix*, Figs. S4 and S5), although to a lesser extent.

We next investigated the circularization efficiency in the absence of residues 350 to 600, including a previously described DNA-binding motif (residues 509 to 557; CtIP_Δ1_) (37), while leaving the N- and C-terminal domains unchanged. At 330 nM (equivalent to 500 tetramers per λ-DNA end), *E*_c_ was 69.9% (Fig. 2*D*), which is very similar to that of wtCtIP and strongly suggests that the DNA-binding motif in the central CtIP region is dispensable for the DNA bridging that induces hybridization and circularization. On removal of residues 165 to 790 to obtain CtIP_Δ2_, a protein similar to the yeast homolog Sae2 (33), *E*_c_ was reduced to 17.4% (Fig. 2*D*). This suggests that compared to CtIP_Δ2_, CtIP_Δ1_ contains residues important for the bridging activity of DNA. All DNA extensions, as well as corresponding STDs and percentiles, are presented in *SI Appendix*, Tables S1–S3.

To explore the importance of the 12-nt overhangs, we performed control experiments with blunt-ended T7-DNA (*SI Appendix*, Fig. S6) as well as a λ-DNA digested with PciI to form a 38.8-kb-long dsDNA with 4-nt complementary overhangs (*SI Appendix*, Fig. S7; details in *Methods*) at CtIP concentrations of 400 nM and 330 nM, respectively (4 µM DNA). No concatemers or circularized DNA molecules were detected for either of the two DNA substrates. This supports the conclusion that the formation of a stable duplex when the overhangs anneal is important for circularization and concatemer formation.

### wtCtIP Condenses Circularized λ-DNA.

**Fig. 3.**
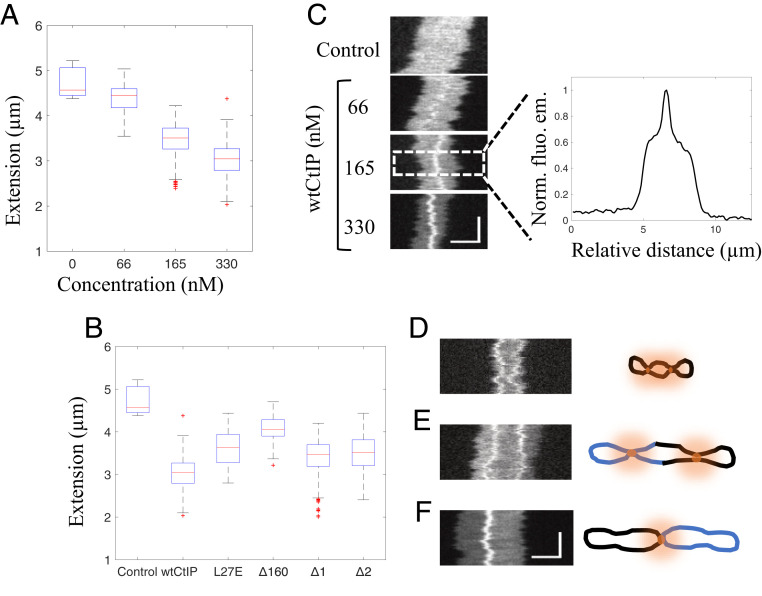
Local compactions reveal accumulation of tetrameric CtIP on the DNA. (*A*) Boxplot of the distribution of extensions for the circular fraction of λ-DNA–wtCtIP complexes at different protein concentrations and a constant DNA concentration of 4 µM bp. (*B*) Boxplot of the distribution of extensions for the circular fraction of λ-DNA–CtIP complexes for different protein derivatives at a protein concentration of 330 nM and a DNA concentration of 4 µM, corresponding to 500 tetramers per λ-DNA end. The blue boxes show the interquartile range (Q2 = 25th percentile, Q3 = 75th percentile) with the median extension (red). Whiskers represent ranges for minimum and maximum, and outliers are represented by red crosses. Datapoints deviating by 1.5 times the interquartile range are considered outliers. (*C*) Representative kymographs of circular λ-DNA molecules at different wtCtIP concentrations. The vertical and horizontal axes correspond to time and molecule extension, respectively. The normalized fluorescence emission graph to the right shows the heterogenous emission along the molecule extension due to the local DNA compaction by wtCtIP. (*D*–*F*) Representative kymographs showing (*D*) a circularized λ-DNA molecule with two dynamic local compactions along the molecule extension (330 nM wtCtIP *n* = 35 [N_tot_ = 3,886], 330 nM CtIP_Δ1_ *n* = 25 [N_tot_ = 2,835]), (*E*) a circularized concatemer of two λ-DNA molecules with two dynamic local compactions along the molecule extension (330 nM wtCtIP *n* = 41 [N_tot_ = 3,886], 330 nM CtIP_Δ1_ *n* = 56 [N_tot_ = 2,835]), and (*F*) two circularized λ-DNA molecules joined through a central static local compaction (330 nM wtCtIP *n* = 44 [N_tot_ = 3,886], 330 nM CtIP_Δ1_ *n* = 64 [N_tot_ = 2,835]). Schematic illustrations to the right visualize the suggested configuration of each complex. Additional kymographs are presented in *SI Appendix*, Figs. S8–S10. The vertical and horizontal scale bars correspond to 3 s and 3 µm, respectively.

We further characterized the circular DNA-protein complexes formed by wtCtIP by determining their extensions in the nanofluidic channels at the different wtCtIP concentrations (Fig. 3*A*). Under the experimental conditions used, circularized λ-DNA molecules without bound protein exhibited a mean extension of 4.7 ± 0.4 µm. On addition of 66 nM wtCtIP to 4 µM bp λ-DNA (100 tetramers per DNA end), the extension of the circles was not affected to a notable extent, yielding a mean extension of 4.4 ± 0.3 µm; however, the average extension was reduced to 3.5 ± 0.3 µm at 165 nM (250 tetramers per DNA end) and further to 3.0 ± 0.3 µm at 330 nM wtCtIP (500 tetramers per DNA end). Thus, at the highest concentration, the average decrease in extension compared to the control was ∼36%.

CtIP_L27E_ and CtIP_Δ160_ were less efficient in the compaction of circularized λ-DNA compared to wtCtIP, showing that tetramerization of CtIP is important for DNA compaction. In contrast, for CtIP_Δ1_ and CtIP_Δ2_, the extension of the circular complexes was similar to that of wtCtIP, showing that the internal regions of CtIP are partially dispensable for DNA compaction (Fig. 3*B*). Thus, while CtIP_Δ2_ is less efficient at circularizing λ-DNA compared to wtCtIP and CtIP_Δ1_, as reflected by the lower *E*_c_, the formed circles were compacted to the same extent. The residues missing in CtIP_Δ2_ (165 to 350 and 600 to 790) are required to stimulate the nucleases of MRN and DNA2 in vitro, and cells expressing the CtIP_Δ2_ variant are consequently deficient in DNA end resection, as measured by single-strand annealing reporter assays (22, 33). As residues 165 to 350 and 600 to 790 of CtIP appear dispensable for DNA compaction, the respective CtIP functions can be separated.

### CtIP Promotes Formation of Higher-Order Circular DNA Structures.

As a next step, we characterized the time-trace images (kymographs) of individual circular DNA-wtCtIP complexes (Fig. 3*C*). The fluorescence intensity varied along the molecule extension, and a bright feature was observed in at least one position along the DNA, particularly at high protein concentrations. At 330 nM wtCtIP (4 µM bp DNA, 500 tetramers per DNA end), this was observed for >60% of the circular DNA-protein complexes detected. The fluorescence emission signal comes from the YOYO-1 DNA dye, indicating a high DNA concentration at this position which suggests local protein-induced DNA condensation. This can be explained by the accumulation of protein along the DNA molecule that locally alters the physical properties of the DNA. Similar local DNA condensation was also observed in the presence of CtIP_Δ1_ and CtIP_Δ2_. For CtIP_ΔL27E_ and CtIP_Δ160_, most circles showed no local condensation. As shown in Fig. 3*C*, the local condensation varied in size, as reflected by the variation in emission intensity, in a manner closely correlated with the reduced overall extension of the circular complexes at increasing concentrations of wtCtIP (Fig. 3*A*).

A few circular complexes with two local condensates along the molecule extension were also observed for wtCtIP and CtIP_Δ1_ (Fig. 3*D* and *SI Appendix*, Fig. S8), implying that the protein can form two separate locally condensed clusters along the same DNA molecule. We also observed DNA-protein complexes with sizes corresponding to two circularized λ-DNA molecules (mainly for wtCtIP and CtIP_Δ1_), with up to two local condensations along the DNA extension (Fig. 3*E* and *SI Appendix*, Fig. S9). This shows that the local condensation is not limited to a single λ-DNA molecule, but also can exist in circularized concatemers. Complexes corresponding to two circularized λ-DNA molecules with an immobile feature at the center of the complex were also observed (Fig. 3*F* and *SI Appendix*, Fig. S10). The apparent static nature of this condensate indicates that two circular λ-DNA molecules have been joined together to form a concatemer of precircularized molecules in which the protein cluster on one or both circles act as an intermolecular bridge to the other one. This shows that locally condensed circles can attract circular DNA molecules to form chain-like structures, suggesting that the DNA-bound protein is to some extent exposed to the surroundings, allowing intermolecular bridging. Such complexes harboring an additional local condensation on one of the circles were also observed (*SI Appendix*, Fig. S11).

### DNA Unfolding Reveals Mobility of Bound CtIP.

**Fig. 4.**
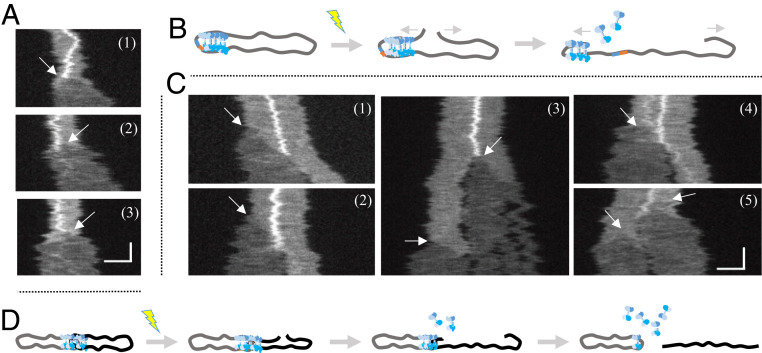
CtIP dissociates from circular complexes upon DNA unfolding. (*A*) Kymographs displaying the unfolding of three circularized λ-DNA–CtIP complexes with local compactions upon photoinduced breaking of the DNA. Arrows indicate where the DSB occurs, which corresponds to the position from where the DNA starts to unfold. Upon unfolding, the local compaction disappears. (*B*) Schematic illustration showing the unfolding event, where the proteins causing a local compaction dissociate as the DNA is unfolding. (*C*) Kymographs of unfolding of five λ-DNA–CtIP complexes, in which two circles are joined through a central static local compaction. The arrows indicate the initiation of DNA unfolding upon photoinduced breaking. As the broken DNA end unfolds, the local compaction disappears once the end has reached the center and the linear molecule is separated from the other circular DNA (clearly discernible in kymographs 1 and 2). The extensive illumination generates additional breaks causing fragmentation of the linearized DNA (3), as well as breaking and unfolding of the remaining circular molecule (4 and 5). (*D*) Schematic illustration showing the unfolding of two circularized λ-DNA–CtIP complexes joined through a local compaction. Upon breaking, the proteins causing the local compaction will dissociate, and the DNA molecules will separate. The vertical and horizontal scale bars correspond to 5 s and 3 µm, respectively.

To further characterize the individual circularized DNA-wtCtIP complexes, we monitored how they respond to DSBs introduced by photoinduced DNA damage that leads to the unfolding of circular DNA (Fig. 1 *A*–*C*) (32). CtIP-bound circular DNA molecules also linearize on DNA breakage, as shown in three representative locally condensed circular λ-DNA–wCtIP complexes (Fig. 4*A*). This observation confirms that the complexes are truly circular DNA with protein bound. As the broken ends separated from each other, the fluorescence emission decreased as a result of the transition from folded circular to linear DNA (30) (Fig. 4*B*). The CtIP-generated local condensation did not prevent the unfolding of DNA. As the DNA opening reached the locally condensed region, the bright feature suddenly disappeared, and the molecule relaxed to its linear conformation with a homogeneous fluorescence emission along the extension. This confirms that the protein is bound to the circularized DNA molecules.

The same procedure was repeated for circular complexes with two connected DNA molecules joined through a wtCtIP cluster (Fig. 4*C*). On introduction of a DSB in one of the circles, only the broken molecule unfolded. As the unfolding end reached the center of the local condensation, we observed a sudden disappearance of the bright feature. Intriguingly, the linearized molecule detached from the intact circular molecule (illustrated in Fig. 4*D*), indicating that the intermolecular DNA bridging is reversible. The immediate drop in fluorescence emission from the bright feature indicates that the proteins likely slide off the DNA at the open end, allowing the DNA to attain its thermodynamically most stable extended conformation.

### CtIP Interacts Nonspecifically with the DNA Sugar-Phosphate Backbone.

**Fig. 5.**
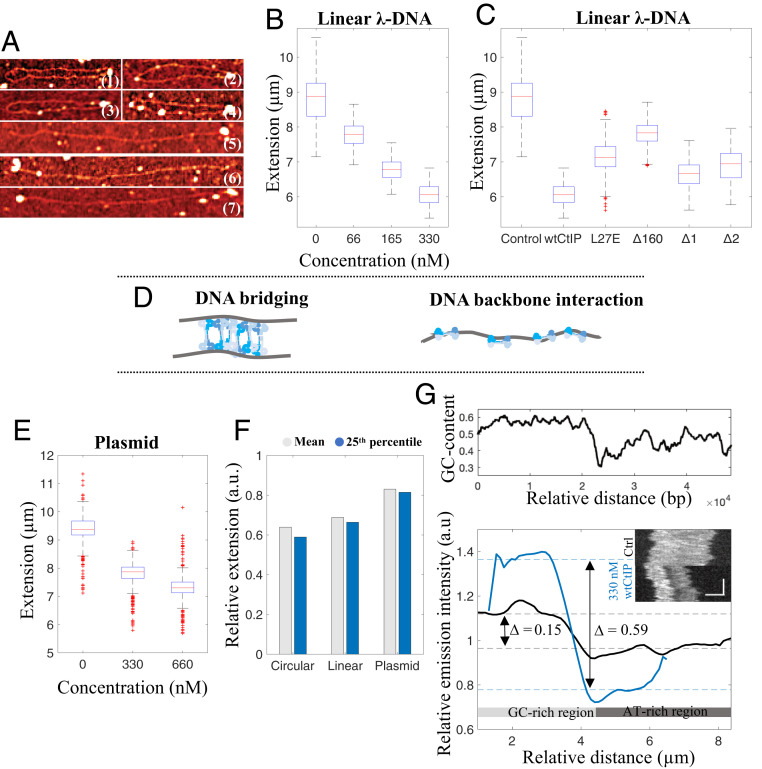
CtIP binds specifically to DNA ends and nonspecifically to the DNA backbone. (*A*) AFM images, showing circular (1 to 4) and linear (5 to 7) DNA-wtCtIP complexes, where features are reflected through the height difference. wtCtIP forms clusters (bright spots) along the contour of both circular and linear DNA molecules as well as at the ends of the linear molecules, suggesting two possible binding modes of CtIP to DNA. Noncropped images are presented in *SI Appendix*, Fig. S9. (*B* and *C*) Boxplots of the distribution of extensions for the nanoconfined linear fraction of λ-DNA–CtIP complexes at different protein concentrations of wtCtIP (*B*) and at a constant protein concentration of 330 nM for different protein variants (*C*). (*D*) Schematic illustration of the proposed DNA-binding mechanisms of CtIP, bridging and binding along the DNA backbone, respectively. (*E*) Boxplot of the extension of a nanoconfined 97-kbp circular plasmid at different concentrations of wtCtIP and a constant total DNA concentration of 4 µM bp. The blue box shows the interquartile range (Q2 = 25th percentile, Q3 = 75th percentile) with the median extension (red). Whiskers represent ranges for minimum and maximum, and outliers are represented by red crosses. Datapoints deviating by 1.5 times of the interquartile range are considered outliers. (*F*) Bar diagram showing the molecule extension relative to control DNA (no protein) for nanoconfined circularized and linear λ-DNA molecules (Circular and Linear, respectively) and for the 97-kbp circular plasmid (Plasmid) at a total DNA concentration of 4 µM bp in the presence of 330 nM wtCtIP. The gray bar represents the mean value for the whole population, whereas the blue bar takes into account only the 25th percentile. The plasmid DNA displays less compaction compared with circularized and linear λ-DNA. (*G*, *Top*) The local GC-content along nanoconfined λ-DNA where a moving average filter has been applied with a step size of 1,000 bp. (*G*, *Bottom*) Emission intensity variation along the molecule extension for bare λ-DNA (black; *n* = 50) and λ-DNA in the presence of 330 nM wtCtIP (blue; *n* = 96), with corresponding representative kymographs as inserts. Color-coded dashed lines mark the median emission values for the first and second halves of the molecule extension, representing the GC-rich (higher emission) and AT-rich (lower emission) regions, respectively. The vertical and horizontal scale bars correspond to 3 s and 3 µm, respectively.

The local condensations in the kymographs in Figs. 3 *C*–*F* and 4 report only indirectly on the DNA–protein interactions, since the protein is not labeled. The association of wtCtIP to dsDNA was confirmed by AFM imaging. A pET plasmid (6.7 kbp) was incubated with wtCtIP and imaged with AFM. Representative images in Fig. 5*A* and *SI Appendix*, Fig. S12 show a clear association between the DNA and protein for both circular and linearized plasmids (27). For the circular molecules (Fig. 5*A*), wtCtIP appears to form clusters randomly along the DNA molecules, confirming the direct interaction between the protein and the sugar-phosphate backbone, independent of free DNA ends. For the linearized DNA, multiple complexes are observed with protein clusters associated along the contour of the DNA molecule. Furthermore, linear complexes were also observed with protein clusters localized at the ends, suggesting specific DNA end interaction, in agreement with previous reports on the CtIP function (27, 37). We also performed AFM experiments for CtIP_L27E_ , which preferentially promotes the formation of intermolecular complexes, which requires binding to free DNA ends. CtIP_L27E_ accumulates mainly at the DNA ends (*SI Appendix*, Fig. S13), demonstrating that it indeed has a high affinity for DNA ends.

### Binding Dynamics of CtIP Depends on DNA Conformation.

When comparing Figs. 1*D* and 2*B*, it is obvious that the DNA extension also decreased for the linear λ-DNA fraction when CtIP was added. In the absence of CtIP, linear λ-DNA had an extension of 8.8 ± 0.6 µm, whereas in the presence of wtCtIP, the DNA extension gradually decreased to 6.1 ± 0.3 µm at 330 nM protein and 4 µM DNA (500 tetramers per DNA end) (Fig. 5*B*). A similar level of compaction was also observed for λ-DNA in the presence of CtIP_Δ1_ and CtIP_Δ2_, while CtIP_L27E_ and CtIP_Δ160_ induced only minor decreases in the extension (Fig. 5*C*). The reduced extension of nanoconfined DNA is caused by binding of CtIP along the DNA backbone as opposed to DNA bridging, similar to other positively charged proteins and ligands. Thus, we propose that CtIP can bind to DNA in two different ways: bridging and binding along the DNA backbone, as schematically illustrated in Fig. 5*D* (6).

We further analyzed the binding of wtCtIP to DNA in a sample containing two large circular plasmids of 62 kbp and 97 kbp (extensions in absence of protein; 6.0 ± 0.3 µm and 9.4 ± 0.5 µm, respectively), which were mostly nicked and therefore topologically relaxed. We then measured the extensions at CtIP tetramer:base pair ratios of 1:50 (330 nM wtCtIP per 4 µM bp) and 1:25 (660 nM wtCtIP per 4 µM bp) (Fig. 5*E* and *SI Appendix*, Fig. S14). The gradual decrease in the extension of both plasmids confirmed that CtIP also compacts relaxed circular DNA. However, comparing the degrees of compaction for the three different DNA templates, circularized λ-DNA, linear λ-DNA, and circular plasmid DNA (97 kbp), revealed that the decrease in extension differed significantly at the same protein:base pair ratio for the different DNA conformations (Fig. 5*F*). While the extension for the circularized λ-DNA molecules decreased by 36.1% on average and the linear ones decreased by 31.2%, the decrease for the relaxed circular plasmids was only 17.0%. The effect is even larger if we consider only the most compacted molecules. For the 25th percentile, the extension of the circularized λ-DNA fraction decreased even further (41.1%; diff = 5.0%), while the effect was smaller for the linear fraction (33.6%; diff = 2.4%) and almost negligible for the circular molecules (18.5%; diff = 1.5%).

In contrast to the single circularized λ-DNA molecules, the linear λ-DNA fraction did not contain local regions with very high emission intensity (*SI Appendix*, Fig. S15). This suggests that the decrease in extension was due to a random distribution of the protein along the DNA rather than to specific cooperative binding, as was seen for the circularized fraction. A possible explanation for this is that the local compactions can be formed only for a topologically closed DNA. However, the kymographs of linear λ-DNA reveal another interesting property of CtIP. For a vast majority of the molecules, the emission intensity varied significantly along the DNA in a way that correlated with the local GC content of λ-DNA (Fig. 5 *G*, *Top*). This pattern resembles the competitive binding of YOYO-1 and the AT-selective drug netropsin, which has been used for optical DNA mapping in nanochannels (38, 39).

To further characterize this sequence-selective binding, all linear kymographs were visually examined, and a consensus emission intensity profile was constructed (details in *Methods*). Intriguingly, for 330 nM CtIP, the difference between the median emission intensity of the GC- and AT-rich halves of λ-DNA was 0.59 a.u. (Fig. 5*G*). The corresponding difference for bare λ-DNA was only 0.15 a.u., suggesting that CtIP binds preferentially to the AT-rich half of λ-DNA and thereby prevents YOYO-1 from binding, resulting in the major emission intensity difference. This observation was consistent for all CtIP derivatives except the monomeric CtIP_Δ160_, in which the difference is only 0.18 a.u., close to bare λ-DNA (*SI Appendix*, Fig. S16 and Table S4).

### *S. cerevisiae* CtIP Homolog Sae2 Bridges DNA.

**Fig. 6.**
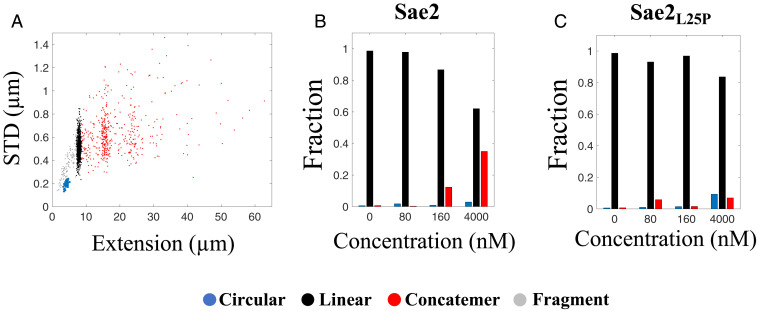
Sae2 promotes concatemer formation of λ-DNA. (*A*) Scatterplot of molecule extension vs. STD for λ-DNA (4 µM bp) incubated with Sae2 (4 µM; *n* = 1,440). Clustering of the datasets was performed to distinguish the circularized λ-DNA molecules (blue) from the full-size linear λ-DNA molecules (black), concatemers (red), and linear fragments (gray). (*B* and *C*) Relative fractions of circular and linear complexes and concatemers for different concentrations of Sae2 (*B*) and Sae2_L25P_ (*C*) at 4 µM bp λ-DNA.

To generalize our results, we next turned to Sae2, the budding yeast homolog of CtIP (14, 24, 40, 41). As described above, Sae2 is a smaller protein than CtIP and resembles the CtIP_Δ2_ mutant in size and sequence. At a 1:1 DNA:protein ratio, phosphorylated Sae2 has the capability of generating both circles and concatemers of λ-DNA (Fig. 6 *A* and *B* and *SI Appendix*, Fig. S17). At concentrations comparable to those for the human proteins presented above, the fractions of circles and concatemers is low, even when compared with CtIP_Δ2_ (Fig. 6*B*). Just as for CtIP, the bridging is dependent on the oligomeric state of the protein. The Sae2 mutant Sae2_L25P_, which can form only dimers, yielded much less circles and concatemers than wild-type Sae2 (Fig. 6*C* and *SI Appendix*, Fig. S18). Both wild-type Sae2 and Sae2_L25P_ reduced the extension of λ-DNA but to a smaller extent than CtIP (*SI Appendix*, Fig. S19). Very few local compactions were observed for circles formed by either Sae2 version, even at the highest concentration. Therefore, while phosphorylated Sae2 is generally capable of compacting and bridging DNA, it is much less efficient than CtIP.

## Discussion

DNA end resection by the MRN complex requires phosphorylation of CtIP (14–16), which paradoxically reduces the binding affinity of CtIP to DNA in traditional gel-based assays. This paradox has raised the question of whether DNA binding and stimulation of DNA end resection are two compatible activities that can be catalyzed by the same population of CtIP molecules or whether DNA binding is even a physiologically important activity of CtIP. To address this discrepancy, we used a nanofluidics-based single-molecule approach to characterize the robust DNA-binding activity of phosphorylated wtCtIP along with a library of CtIP variants, with the aim of providing insight into how CtIP can play an important role in structuring two proximal DNA ends in DSB repair.

We demonstrate that wtCtIP interacts with DNA in a way that causes the 12-nt single-stranded overhangs of λ-DNA to hybridize. Efficient bridging also leads to local condensation of DNA along the sugar-phosphate backbone. The formation of circles with local compactions is unique for CtIP and was not observed with, e.g., RAD51, another hallmark HR protein that forms extended filaments in nanofluidic channels (42). Circularization by hybridization was previously observed for the NC chaperone from the HIV-1 virus, but no local compaction was detected (7). The formation of circles suggests that wtCtIP forms intramolecular bridges on the DNA molecule that bring the free ends into close proximity and thereby increases the probability of self-hybridization. The condensation is much more efficient for λ-DNA that underwent circularization catalyzed by CtIP compared to DNA that was circular before the addition of CtIP, suggesting that the interaction involves loading of protein onto the free DNA ends. This agrees with the AFM analysis in which protein complexes were observed at the ends of linear DNA molecules.

The absence of local condensates along linear DNA stretched in nanofluidic channels suggests that this particular feature occurs only in conjunction with circularization of λ-DNA, further supporting the intramolecular bridging ability of CtIP. We propose that such DNA bridging by CtIP may provide a stimulatory annealing function in MMEJ or help coordinate the DNA end resection of two broken DNA ends at the onset of HR (18). Accumulation of protein at the DNA ends also occurs for the 4-nt overhangs, but no permanent hybridization is observed owing to the less stable duplex formed.

It is interesting to consider the fact that some CtIP variants promote intramolecular hybridization while others, particularly the dimeric CtIP_L27E_, mainly promote the formation of concatemers. Our data suggest that the difference is a kinetic effect. For wtCtIP and CtIP_Δ1_, intramolecular hybridization is faster than concatemerization, and thus fewer concatemers are formed. However, for CtIP_L27E_, which is unable to bridge DNA, intermolecular hybridization is promoted, resulting in concatemers. This was confirmed by experiments at different total concentrations of DNA and protein for CtIP_L27E_, in which a lower total concentration led to the formation of fewer concatemers. This agrees with a previous study in which CtIP_L27E_ allowed for MMEJ in wtCtIP-depleted cells (15).

The significant decrease in circularization efficiency for the structural mutants CtIP_L27E_ and CtIP_Δ160_ suggests that the previously reported tetrameric structure (27) of wtCtIP imposes a key role in bridging DNA. You et al. (37) reported that modifying a minimal damage recruitment (DR) motif (amino acids 509 to 557) in CtIP exerted a down-regulating effect on the damage recruitment capacity, possibly due to the reduced DNA-binding ability of the protein. In contrast to this, complete removal of this DR motif had no effect on the circularization efficiency of λ-DNA compared with wtCtIP, implying that the structuring of DNA ends by CtIP is dependent on other parts of the protein. Wilkinson et al. (27) reported that wtCtIP attains a dumbbell-shaped tetrameric structure, where four C-terminal domains form two globular DNA-binding units, while the N-terminal domains allow for tetramerization. Such a dumbbell-shape, which is disrupted in the dimeric and monomeric forms, closely agrees with our observation on DNA bridging by CtIP. The dumbbell shape is expected to be conserved in case of the two internal deletion mutants CtIP_Δ1_ and CtIP_Δ2_, but only CtIP_Δ1_ retains the same circularization efficiency as wtCtIP.

Assuming that the circularization is directly linked to bridging, it is not possible to directly determine whether this difference is due to specific interactions in the deleted regions or to changes in the physical dimensions of the protein. Our data support a picture in which the shorter length of CtIP_Δ2_ makes it more difficult to bring two distant DNA segments in close proximity. A shorter dumbbell must hold the two highly negatively charged DNA double-helices closer than the full-length wtCtIP, which is energetically unfavorable. This hypothesis is supported by the observation that CtIP_Δ2_ causes a similar decrease in extension of linear DNA as CtIP_Δ1_ and wtCtIP, which indicates that the binding affinity to DNA is similar. A decreased bridging capability is consistent with biochemical studies on HR in vivo, in which the DNA repair function is dramatically impaired in the presence of CtIP_Δ2_ compared to wtCtIP and CtIP_Δ1_ (33).

Intriguingly, most CtIP derivatives studied (except CtIP_Δ160_) seem to preferably bind to AT-rich regions along DNA. The deviant behavior of CtIP_Δ160_ potentially may be explained by its weaker binding to DNA. While the AT selectivity might be less relevant in vivo, since CtIP preferentially acts at DSBs, it might be important for understanding the binding of CtIP to DNA. A preferred binding to AT-rich DNA might reflect the need for CtIP to partially open the DNA helix to facilitate binding, which would be easier in AT-rich regions that are less stable and more prone to melting (43). In addition, the persistence length of DNA increases by up to 20% as the GC content of DNA increases, which also might contribute to the observed selectivity for the softer AT-rich regions (44).

A major benefit of the nanofluidics methodology is the possibility of studying individual DNA-protein complexes without tethering of the DNA ends or immobilizing the complexes on a surface. The locally compacted circular λ-DNA molecules are examples of such structures, where the condensation indicates local accumulation of CtIP and DNA. This local condensation disappears on linearization of the complex, which indicates that the protein binds in a dynamic manner to the DNA backbone and falls off as the free DNA end approaches, when the DNA is unfolding into its linear conformation. Furthermore, the bridging of circular complexes through such a local condensation suggests that the protein is to some extent exposed to the surroundings when associated with DNA. This indicates that CtIP can potentially bind to a damage site and keep the DNA ends close while allowing recruitment of additional factors, such as the MRN complex, for further processing of the ends and facilitation of DSB repair.

The yeast homolog Sae2 is smaller than the human CtIP and consequently much less efficient in DNA bridging. DNA hybridization was observed only at very high Sae2 concentrations. Whether this observation is related to the very low frequency of MMEJ in budding yeast remains to be determined (45).

To conclude, our data suggest two different binding modes of the CtIP protein. The first mode results in the accumulation of CtIP clusters on the DNA, causing local condensation, which can explain the potential of CtIP to hold broken DNA ends together during HR via DNA bridging. This local condensation seems to be promoted by loading of protein on the free DNA ends. The second mode is a general affinity of CtIP for the DNA backbone, causing a decrease in extension that is in agreement with association of a polycationic protein to the backbone of DNA (6). This second mode of binding occurs preferentially in AT-rich regions of DNA. Our results show that wtCtIP in its phosphorylated form is able to stably interact with DNA and bring free DNA ends into close proximity to allow annealing. This further suggests that the biologically active phosphorylated wtCtIP is indeed able to both promote MRN nuclease activity and perform DNA bridging, which may be important for both HR and MMEJ.

## Methods

### Protein Purification.

Recombinant phosphorylated CtIP protein, the different phosphorylated CtIP derivatives, and human MRN were purified from *Spodoptera frugiperda* 9 (*Sf*9) cells using affinity chromatography as described elsewhere (16, 33). Phosphorylated yeast Sae2 and Sae2_L25P_ were purified from *Sf*9 cells by affinity chromatography, as described elsewhere (35).

### Single-Molecule Nanofluidics.

The various CtIP derivatives were mixed with sticky-ended λ-phage DNA (48,502 bp; Roche) at the different ratios indicated in each experiment in buffer I (10 mM Tris⋅HCl pH 7.6, 10 mM NaCl, and 5 mM DTT). Incubation of 330 nM CtIP per DNA end was used for most experiments, where the DNA bp concentration was set to 4 µM. This corresponds to an approximate ratio of one CtIP tetramer to 50 bp DNA (500 tetramers per end). The DNA-protein complexes were confined in the nanofluidic channels and imaged with epifluorescence microscopy. Further details are provided in *SI Appendix*, *Extended Materials and Methods*.

### Data Analysis.

The collected images were analyzed using a custom-written MATLAB-interfaced software. The circular DNA-CtIP complexes were distinguished from the other populations of molecules in the scatterplots with a clustering approach using the free statistical software R. Further details can be found in *SI Appendix*, *Extended Materials and Methods*.

### AFM.

Four µM bp of pET-plasmid was incubated with 330 nM wtCtIP or CtIP_L27E_ (one tetramer [wtCtIP] or two dimers [CtIP_L27E_ dimers] per 50 bp DNA), equivalent to that of 500 CtIP tetramers per λ-DNA end in the nanofluidic experiments. The AFM images were acquired in air using an NTEGRA Prima scanning probe microscope, operating in a tapping mode. Further details can be found in *SI Appendix*, *Extended Materials and Methods*.

### Gel-Based Assays.

Details of the λ-phosphatase treatment, the electrophoretic mobility shift assay and the nuclease assay are provided in *SI Appendix*, *Extended Materials and Methods*.

## Supplementary Material

Supplementary File

## Data Availability

All pertinent data are provided in the main text and *SI Appendix*.
