# Supplementary material for: Phosphorylated CtIP bridges DNA to promote annealing of broken ends
Source: Proc Natl Acad Sci U S A. 2020 Aug 19;117(35):21403–12. doi: 10.1073/pnas.2008645117 (PMC7474685; doi:10.1073/pnas.2008645117)
Supplement: Supplementary File [file pnas.2008645117.sapp.pdf]

PNAS
www.pnas.org

Supplementary Information for

Phosphorylated CtIP bridges DNA to promote annealing of broken ends

Robin Öz, Sean M. Howard, Rajhans Sharma, Hanna Törnkvist, Ilaria Ceppi, Sriram KK, Erik Kristiansson, Petr Cejka, Fredrik Westerlund

Fredrik Westerlund
Email: fredrik.westerlund@chalmers.se

This PDF file includes:

Extended Materials and Methods
Figures S1 to S19
Tables S1 to S4
SI References

www.pnas.org/cgi/doi/10.1073/pnas.2008645117

Extended Materials and Methods

Single Molecule Nanofluidics

The various CtIP derivatives were mixed with sticky-ended λ-phage DNA (48502 bp, Roche) at the different ratios indicated in each experiment in buffer I (10 mM Tris-HCl pH 7.6, 10 mM NaCl and 5 mM DTT). Incubation of 330 nM CtIP per DNA-end was used for most experiments, where the DNA base pair concentration was set to 4 µM. This corresponds to an approximate ratio of one CtIP tetramer to 50 bp DNA. For the structural mutants, the ratio of protein to DNA was multiplied by two for $CtIP_{L27E}$ (dimer) and by four for $CtIP_{\Delta160}$ (monomer). For the plasmid sample, 4 µM DNA was mixed with 330 nM and 660 nM wtCtIP respectively. For blunt-ended T7-DNA (39936 bp, Mabion), 4 µM DNA was mixed with 400 nM wtCtIP, corresponding to 500 tetramers per DNA end. 4µM of PciI-digested λ-DNA was mixed with 330 nM wtCtIP and $CtIP_{L27E}$. The Sae2-derivatives were mixed with DNA at a ratio of 1 protein to 1, 25 and 50 base pairs respectively. The samples were incubated at 37°C for one hour followed by addition of the bis-intercalating fluorescent dye YOYO-1 at a ratio of 1 dye molecule per 5 bp DNA and incubation for additional 10 minutes at 25°C. The samples were diluted to a final concentration of 1 µM DNA base pair and supplemented with sodium dodecyl sulphate (SDS) at a final concentration of 0.05% (w/w) to obtain buffer A (10 mM Tris-HCl pH 7.6, 10 mM NaCl, 5 mM DTT, 0.05% SDS), which was used as analysis buffer in all nanofluidic experiments. SDS was included to reduce sticking of the DNA-protein complexes to the channel walls.

The silicon dioxide based nanofluidic chips with channel dimensions of 150 x 100 nm^2 were fabricated as described elsewhere[1] and used for all single-molecule experiments. The nanofluidic chip was equilibrated with buffer A at 25°C prior to loading of the YOYO-1 stained DNA-CtIP sample in one of the four reservoirs. Pressurized nitrogen gas was used to control the liquid flow within the nanofluidic chip. By manipulating the applied pressure on each individual reservoir, the pre-formed DNA-CtIP complexes were pre-concentrated in the microchannel at the entrance of the nanochannels, before being driven in to the nanochannels, to simultaneously image as many individual DNA-CtIP complexes as possible. The confined DNA-CtIP complexes were visualized on an inverted fluorescence microscope (Zeiss AxioObserver.Z1) equipped with a 100x oil

immersion objective (NA = 1.46), a Colibri 7 LED light source (Zeiss) and an sCMOS Prime 95B camera (Photometrics). Blue light (469/38 nm) was used to excite the sample and the emission was passed through a single band pass filter (530/30 nm) before reaching the detector. To determine the extension of the DNA-CtIP complexes, 50 frames were recorded for each molecule, with a frame rate of 0.13 s/frame. For visualizing DNA unfolding, up to 300 frames were recorded at the same frame rate.

Data analysis

The collected images were analyzed using a custom-written MATLAB-interfaced software, where each individual DNA-CtIP complex was detected. The recorded image stacks for each detected complex were converted to a kymograph, from which the extensions and corresponding standard deviations were calculated[2]. Complexes, which were found to break during the course of recording were excluded from the size analysis. Size distribution histograms and standard deviation scatterplots were created using MATLAB.

To distinguish the circular DNA-CtIP complexes from the other populations of molecules, a clustering approach was employed using the free statistical software R. The molecule extensions and the associated internal standard deviations were log-transformed, followed by applying hierarchical clustering using Euclidian distance metric and employing Ward's minimum variance method to specify the dissimilarity of clusters. The complexes found in the cluster corresponding to the circular fraction are highlighted in the respective scatter plots (molecule extension vs standard deviation). A similar approach was used to distinguish the full-size linear λ-DNA-CtIP complex. In this way the more scattered full-size linear DNA-CtIP fraction formed distinct clusters, from which we could determine the number of intact λ-DNAs as well as the mean extension and the corresponding standard deviation. In order to account for all full-size linear complexes in the population, which display higher lateral flexibility than the circular complexes, the sizes of the partially clustered linear complexes were plotted in a histogram to which a normal probability distribution function was fitted. The resulting mean extension (μ) and standard deviation (σ) were

obtained from the fit. All molecules with an extension of $\mu \pm 2\sigma$ were considered to be full-size linear λ-DNA-CtIP complexes, independent of standard deviation.

The circularization efficiency (E_c) was calculated from the number of circles detected, compared to the total number of circular molecules, full-length λ-DNA molecules and longer complexes (corresponding to blue, black and red fractions, respectively, in the scatterplots and histograms). The level of concatemerization was calculated from the number of molecules longer than that of one linear full-length λ-DNA molecule, compared to the total number of molecules corresponding to circular, linear full-length and larger molecules. DNA fragments were not included in the calculations.

For the sequence specificity analysis, kymographs of linear DNA molecules were picked based on their fluorescence emission signal-intensity profiles. Molecules with profiles resembling the GC-content profile of λ-DNA, were combined to form a consensus intensity-profile[3]. The median relative intensity of the GC- and AT-rich regions (first and second half of the intensity profile respectively) were determined and the difference was calculated for each sample.

Atomic force microscopy (AFM)

4 μM bp of a pET-plasmid was incubated with 330 nM wtCtIP or $CtIP_{L27E}$. This concentration was set to obtain a ratio of one wtCtIP tetramer or two $CtIP_{L27E}$ dimers per 50 bp DNA, equivalent to that of 500 CtIP tetramers per λ-DNA-end in the nanofluidic experiments. The DNA and protein were mixed in buffer I for one hour at 37°C before depositing 15 μl of the sample on a mica surface. The DNA-CtIP complexes were allowed to adsorb to the surface for 10 minutes at room temperature, followed by rinsing the mica with ultrapure MilliQ water. Pressurized nitrogen was used to dry the surface. The AFM images were acquired in air using an NTEGRA Prima scanning probe microscope, operating in tapping mode with golden silicon probes (force constant 1.45-15.1, resonance frequency 87-203 kHz). The scanning rate was 1 Hz. The resulting images were treated in the open source software Gwyddion[4].

λ-phosphatase treatment

Phosphorylated wtCIP was dephosphorylated by mixing 1 µg of the protein with 200 U λ-phosphatase (NEB) in 1x PMP buffer (NEB) and 1mM $MnCl_2$. The total volume was adjusted to 20 µl by addition of water. A mock-reaction was run simultaneously, where the λ-phosphatase was replaced by water. The samples were incubated at 30°C for 15 minutes, followed by separation on a denaturing polyacrylamide gel.

Electrophoretic mobility shift assay

When linear DNA was used, pUC19 was linearized with EcoRI (New England Biolabs) according to manufacturer's instructions. Both for linear and circular DNA, the reactions (15 µl volume) were performed in binding buffer containing 25 mM Tris-acetate (pH 7.5 at 25 °C), 1 mM dithiothreitol (DTT), 1 mM ATP, 0.25 mg/ml BSA (New England Biolabs) and 100 ng DNA substrate. For experiments in the presence of EDTA, 2 mM EDTA was used. For experiments in the presence of magnesium, the binding buffer was supplemented with 5 mM magnesium-acetate and 1 mM ATPγS. Topoisomerase I was added to relax the circular DNA, and the reaction was incubated for 10 min at 37°C. After the addition of wtCtIP, the reactions were incubated on ice for 30 min. Loading dye (50% glycerol, bromophenol blue) was then added and the products were separated by 0.6% agarose gel electrophoresis in Tris-Acetate-EDTA (TAE) buffer. The electrophoresis was carried out in a cold room at 4°C and the DNA was visualized by staining with GelRed (Biotium).

Nuclease assay

To prepare the quadruple blocked 70-bp long DNA substrate, PC210 and PC211 oligonucleotides were used, as described previously[5]. Briefly, PC210 was labeled at the 5'-end by T4 polynucleotide kinase (New England Biolabs) and [γ-32P] ATP (Perkin Elmer) according to the manufacturer's instructions. Unincorporated nucleotides were removed using Micro Bio-Spin P-30 Tris chromatography columns (Biorad)[6].

Endonuclease assays (15 µl volume) were performed in nuclease buffer containing 25 mM Tris-HCl pH 7.5, 5 mM magnesium acetate, 1 mM manganese acetate, 1 mM dithiothreitol (DTT),

1 mM ATP, 0.25 mg/ml BSA (New England Biolabs), 1 mM phosphoenolpyruvate (Sigma), 80 U/ml pyruvate kinase (Sigma), and 1 nM oligonucleotide-based DNA substrate (in molecules)[6]. The reactions were supplemented with 15 nM streptavidin (Sigma) and incubated for 5 min at room temperature to block the biotinylated ends of the DNA substrates. The recombinant proteins were then added to the reactions on ice and samples were incubated at 37°C for 30 min. After the addition of 0.5 µl of 0.5 M EDTA and 1 µl Proteinase K (19 mg/ml, Roche), reactions were stopped by incubation at 50°C for 30 min. Finally, 16.5 µl loading buffer (5% formamide, 20 mM EDTA, bromophenol blue) was added to all samples and the products were separated on 15% polyacrylamide denaturing urea gels (19:1 acrylamide-bisacrylamide, Bio-Rad), as described elsewhere[6]. The gels were fixed in fixing solution (40% methanol, 10% acetic acid, 5% glycerol) for 30 min at room temperature and dried on a 3MM Chr paper (Whatman). The dried gels were exposed to storage phosphor screen (GE Healthcare) and scanned by a Typhoon Phosphor Imager (FLA 9500, GE Healthcare).

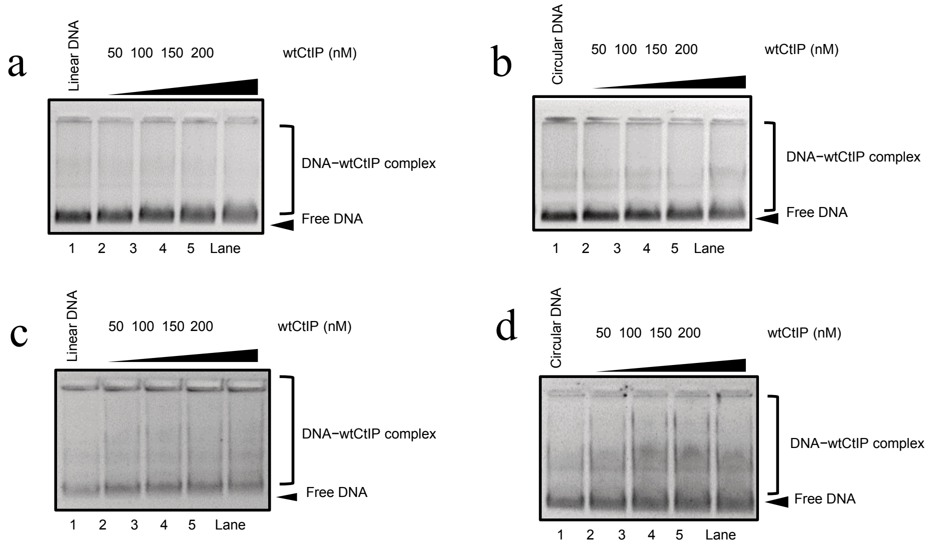

Fig. S1. EMSA-gels of a blunt-ended linear **(a, c)** and circular **(b, d)** 2.9 kbp pUC19 plasmid at different concentrations of wtCtIP in the presence of EDTA **(a, b)** and magnesium **(c, d)**. Only a slight shift is observed for the circular DNA, which may suggest higher affinity of wtCtIP for circular compared to linear DNA.

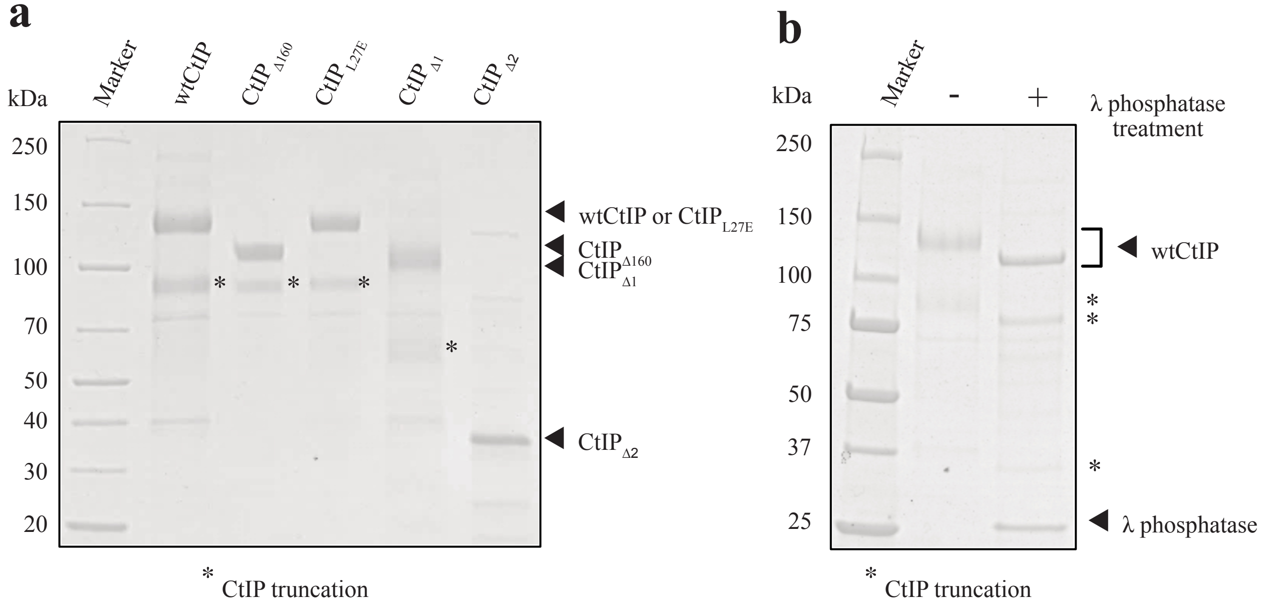

Fig. S2. (a) A poly-acrylamide gel showing the different CtIP derivatives used in this study. Truncations as result of inevitable protein degradation over time are marked with an asterisk. **(b)** λ-phosphatase treatment of wtCtIP. The increased electromobility upon dephosphorylation by λ-phosphatase indicates that the CtIP protein is phosphorylated.

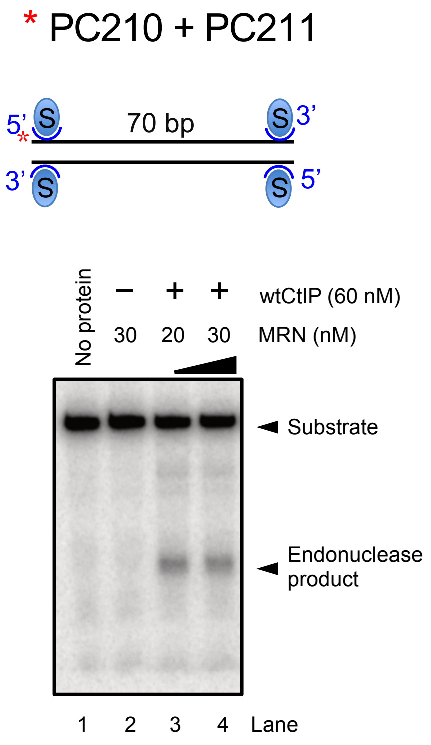

Fig. S3. Representative endonuclease assay with various concentrations of MRN and wtCtIP on a 5' end-labeled 70 bp-long dsDNA blocked at both ends with streptavidin. Red asterisk indicates the position of the labelling. wtCtIP enhances the nuclease activity of MRN.

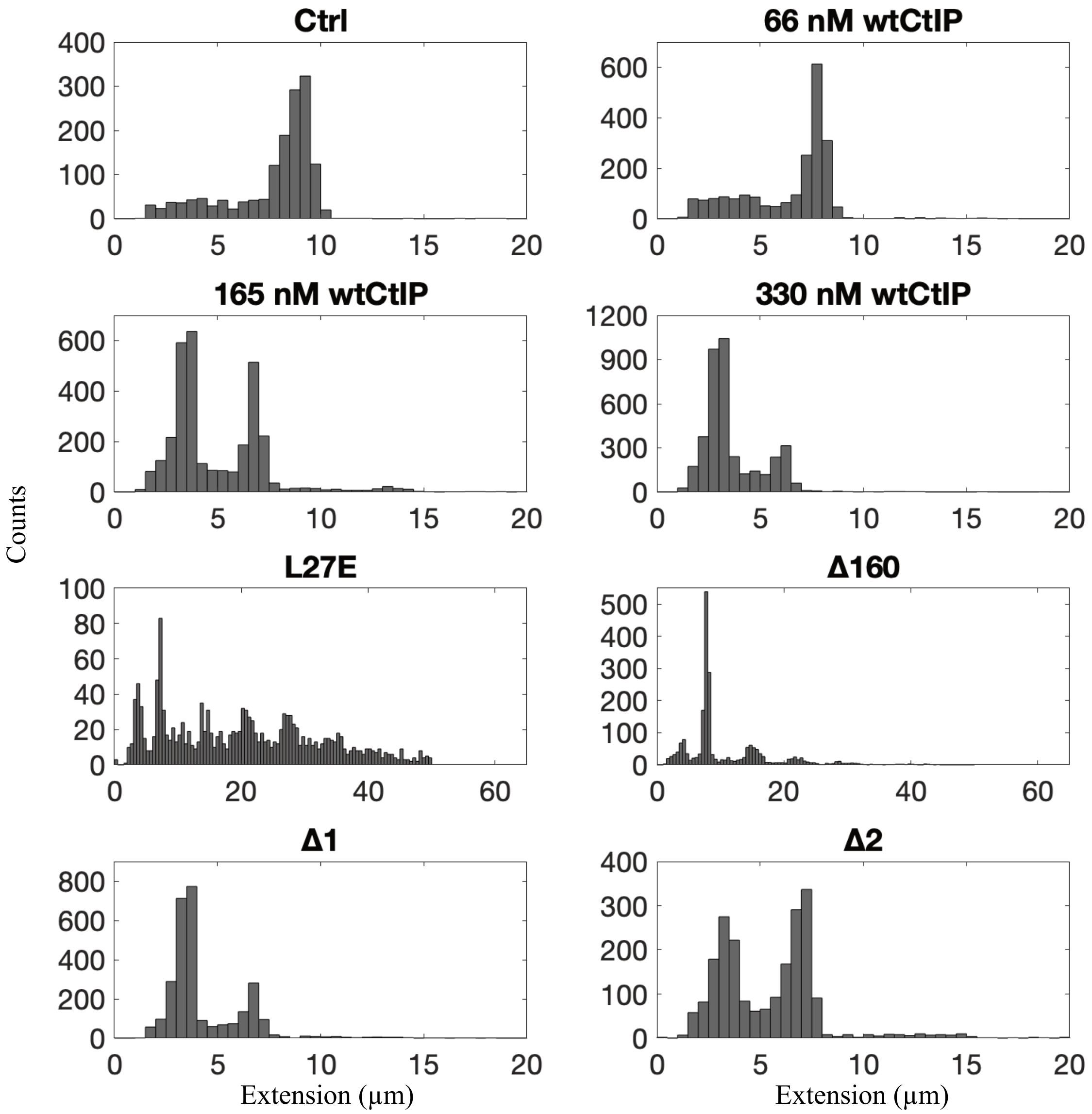

Fig. S4. Histograms of the distribution of DNA extensions for 4 μM λ-DNA (bp) incubated with different concentrations of wtCtIP, as well as with different CtIP variants at a concentration of 330 nM, equivalent to 500 tetramers per DNA end. Bin-size 0.5 μm.

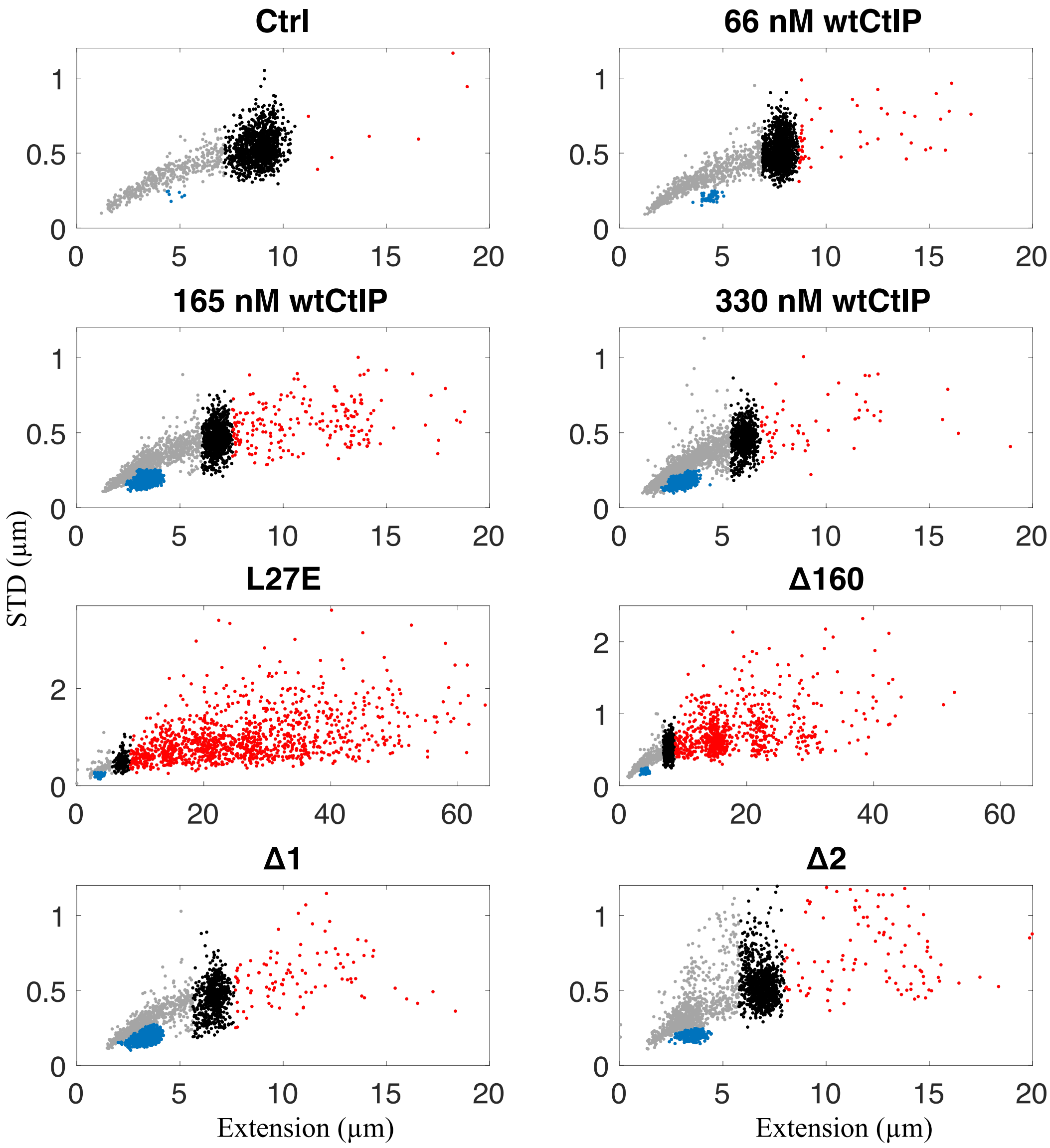

Fig. S5. Scatter plots of molecule extensions vs. STD for 4 μM λ-DNA (bp) incubated with different concentrations of wtCtIP, as well as with different CtIP variants at a concentration of 330 nM, equivalent to 500 tetramers per DNA end. Clustering of the datasets was performed to distinguish the circular molecules (blue) from the full-size linear λ-DNA molecules (black), concatemers (red) and fragments (grey).

Table. S1. Mean and median molecule extensions of circularized λ-DNA-CtIP complexes with corresponding values for 25th and 75th percentiles for each CtIP derivative. Circles were detected by hierarchical clustering, except for the control sample (Ctrl), where molecules were detected manually. The proteins were incubated with λ-DNA at a concentration of 4 μM bp.

	**Ctrl λ-DNA**	**wtCtIP 66 nM**	**wtCtIP 165 nM**	**wtCtIP 330 nM**	**L27E 330 nM**	**Δ160 330 nM**	**Δ1 330 nM**	**Δ2 330 nM**
Mean (μm)	4.74	4.40	3.48	3.03	3.60	4.05	3.42	3.51
Median (μm)	4.57	4.45	3.51	3.04	3.63	4.07	3.47	3.52
25th percentile (μm)	4.45	4.18	3.26	2.79	3.27	3.90	3.19	3.21
75th percentile (μm)	5.06	4.60	3.73	3.27	3.94	4.29	3.70	3.81
N	7	53	1112	1713	78	99	1546	221

Table. S2. Mean and median molecule extensions of linear λ-DNA-CtIP complexes with corresponding values for 25th and 75th percentiles for each CtIP derivative. The proteins were incubated with λ-DNA at a concentration of 4 μM bp.

	**Ctrl λ-DNA**	**wtCtIP 66 nM**	**wtCtIP 165 nM**	**wtCtIP 330 nM**	**L27E 330 nM**	**Δ160 330 nM**	**Δ1 330 nM**	**Δ2 330 nM**
Mean (μm)	8.79	7.77	6.78	6.05	7.13	7.81	6.63	6.89
Median (μm)	8.88	7.79	6.78	6.06	7.13	7.83	6.66	6.95
25th percentile (μm)	8.31	7.53	6.56	5.84	6.86	7.60	6.38	6.55
75th percentile (μm)	9.26	8.03	7.00	6.29	7.44	8.05	6.91	7.24
N	1101	1230	924	640	198	1032	569	930

Tab. S3. Mean and median molecule extensions of DNA-CtIP complexes for two plasmids of different sizes with corresponding values for 25th and 75th percentiles for different CtIP concentrations. The plasmids were detected by hierarchical clustering. The total DNA concentration in each sample was 4 µM (bp).

	**Ctrl**		**330 nM wtCtIP**		**660 nM wtCtIP**	
	**62 kbp**	**97 kbp**	**62 kbp**	**97 kbp**	**62 kbp**	**97 kbp**
Mean (µm)	6.04	9.38	4.97	7.79	4.72	7.32
Median (µm)	6.03	9.37	5.10	7.87	4.71	7.30
25th percentile (µm)	5.88	9.18	4.79	7.64	4.61	7.13
75th percentile (µm)	6.25	9.67	5.20	8.04	4.85	7.51
N	609	757	402	613	552	762

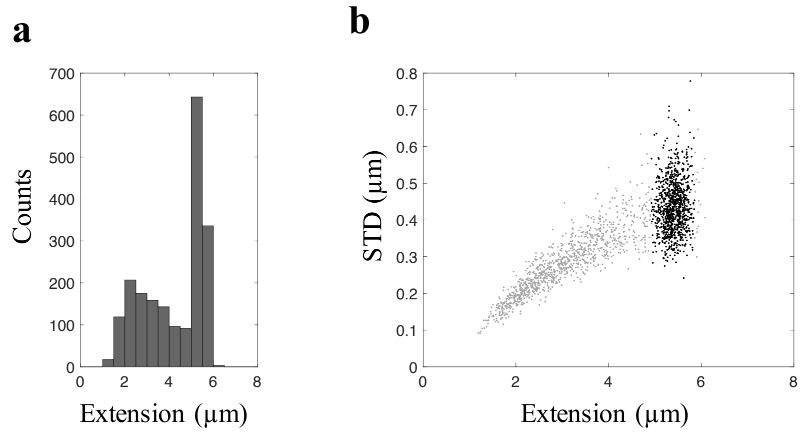

Fig. S6. **(a)** Histogram of the distribution of T7-DNA extensions in the presence of wtCtIP at a concentration corresponding to 500 tetramers per DNA end, DNA concentration 4 μM bp. Bin-size 0.5 μm. **(b)** The corresponding scatter plot of molecule extension vs. STD (N = 1990). Clustering of the datasets was performed to distinguish the full-size linear λ-DNA molecules from fragments (grey). No circles or concatemers are identified.

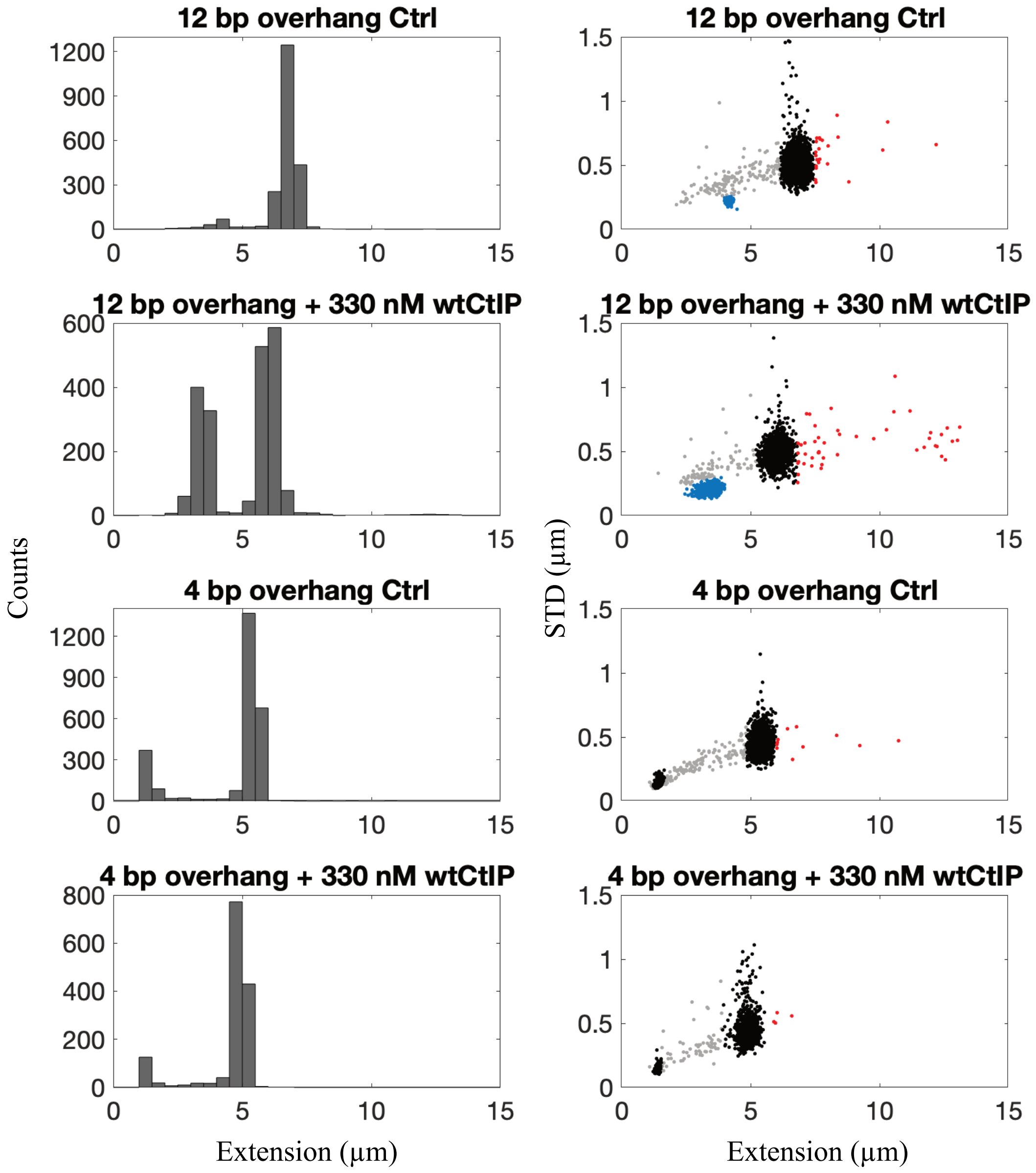

Fig. S7. Size histograms and scatter plots for 4 μM (bp) λ-DNA (12 nt complementary overhangs) and PciI-digested λ-DNA (4 nt complementary overhangs) in the absence and presence of 330 nM wtCtIP. Clustering of the datasets was performed to distinguish the full-size linear molecules from fragments (grey). No circles (blue) were identified for the PciI-digested λ-DNA in contrast to the full-length λ-DNA controls under the same buffer conditions. Concatemers are marked in red.

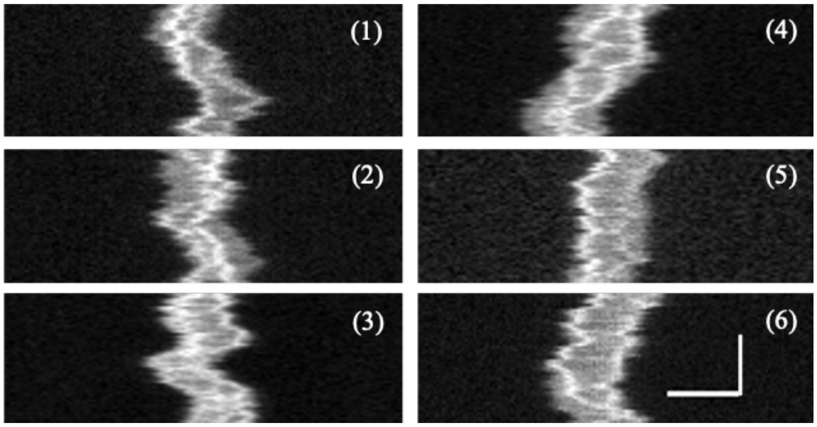

Fig. S8. Representative kymographs of circularized λ-DNA molecules with two local compactions along the molecule extension (similar to **Fig. 3d**), mainly observed for wtCtIP, $CtIP_{\Delta 1}$ and $CtIP_{\Delta 2}$ at 330 nM and 4 μM DNA (bp). The vertical and horizontal scale-bars correspond to 3 s and 3 μm, respectively.

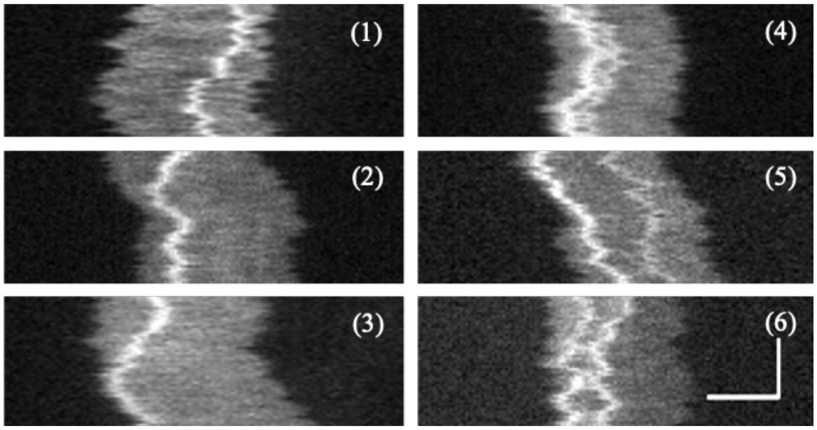

Fig. S9. Representative kymographs of circularized concatemers of two λ-DNA molecules with one (1-3) and two (4-6) dynamic local compactions along the molecule extension (similar to **Fig. 3e**), mainly observed for wtCtIP and $CtIP_{\Delta 1}$ at 330 nM and 4 μM DNA (bp). The vertical and horizontal scale-bars correspond to 3 s and 3 μm, respectively.

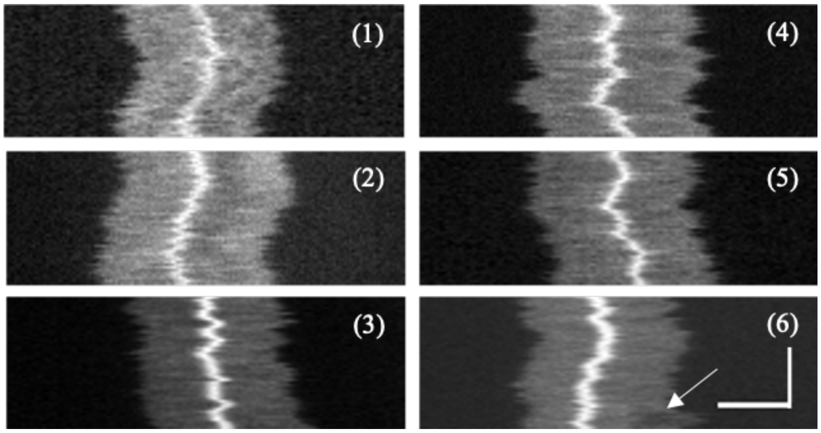

Fig. S10. Representative kymographs of two circularized λ-DNA molecules joined through a central static local compaction (similar to **Fig. 3f**), mainly observed for wtCtIP and $CtIP_{\Delta 1}$ at 330 nM and 4 μM DNA (bp). The arrow in kymograph (6) shows the initiation of DNA unfolding upon photo-induced double-stranded break. The vertical and horizontal scale-bars correspond to 3 s and 3 μm, respectively.

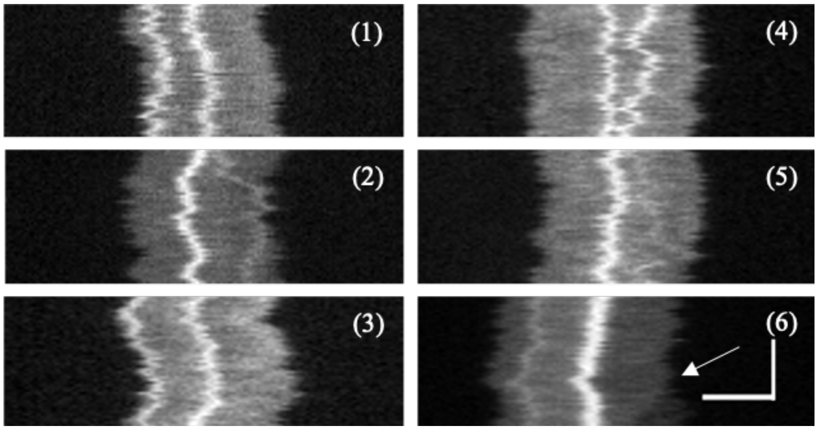

Fig. S11. Representative kymographs of two circularized λ-DNA molecules joined through a central static local compaction (similar to Fig. **3f**), with an additional dynamic local compaction on one of the circles, mainly observed for wtCtIP and $CtIP_{\Delta 1}$ at 330 nM and 4 μM DNA (bp). The arrow in kymograph (6) shows the initiation of DNA unfolding upon a photo-induced double-stranded break. The vertical and horizontal scale-bars correspond to 3 s and 3 μm, respectively.

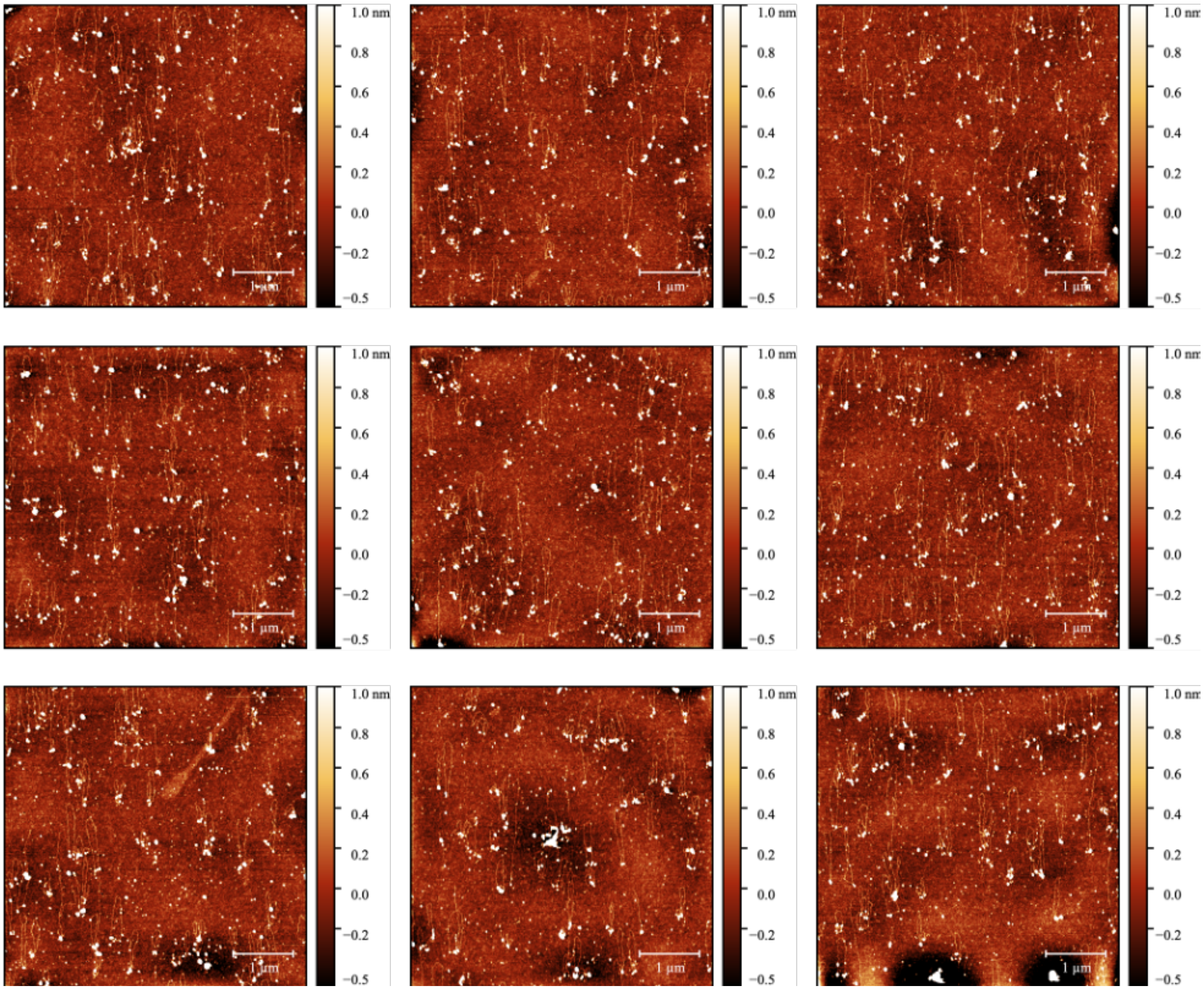

Fig. S12. AFM images of 330 nM wtCtIP incubated with 4 μM (bp) circular and linearized pET plasmids (6.7 kbp). Circular and linear DNA-wtCtIP complexes are observed on a 5 μm x 5 μm mica-surface, where features are reflected through the height difference, along with the false-color gradient scale, indicating the relative height in nm. wtCtIP forms clusters (bright spots) along the contour of both circular and linear DNA molecules as well as at the ends of the linear molecules. Scale-bars correspond to 1 μm.

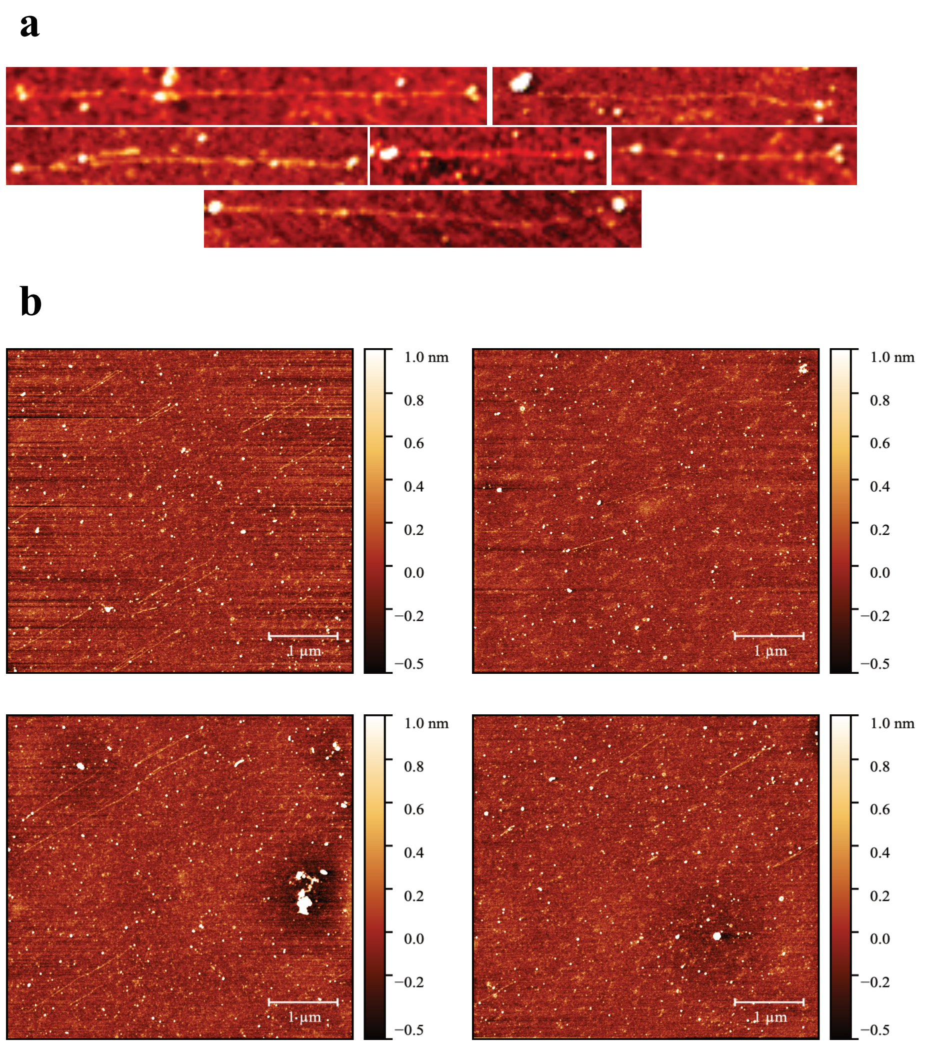

Fig. S13. (a) AFM images of 330 nM CtIP$_{L27E}$ incubated with 4 µM (bp) linearized pET plasmid (6.7 kbp), showing DNA-CtIP$_{L27E}$ complexes, where features are reflected through the height difference. CtIP$_{L27E}$ mainly forms clusters (bright spots) at the ends of the sticky-ended linear molecules. **(b)** Non-cropped images of DNA-CtIP$_{L27E}$ complexes on a 5 µm x 5 µm mica-surface along with the false-color gradient scale, indicating the relative height in nm. Scale-bars correspond to 1 µm.

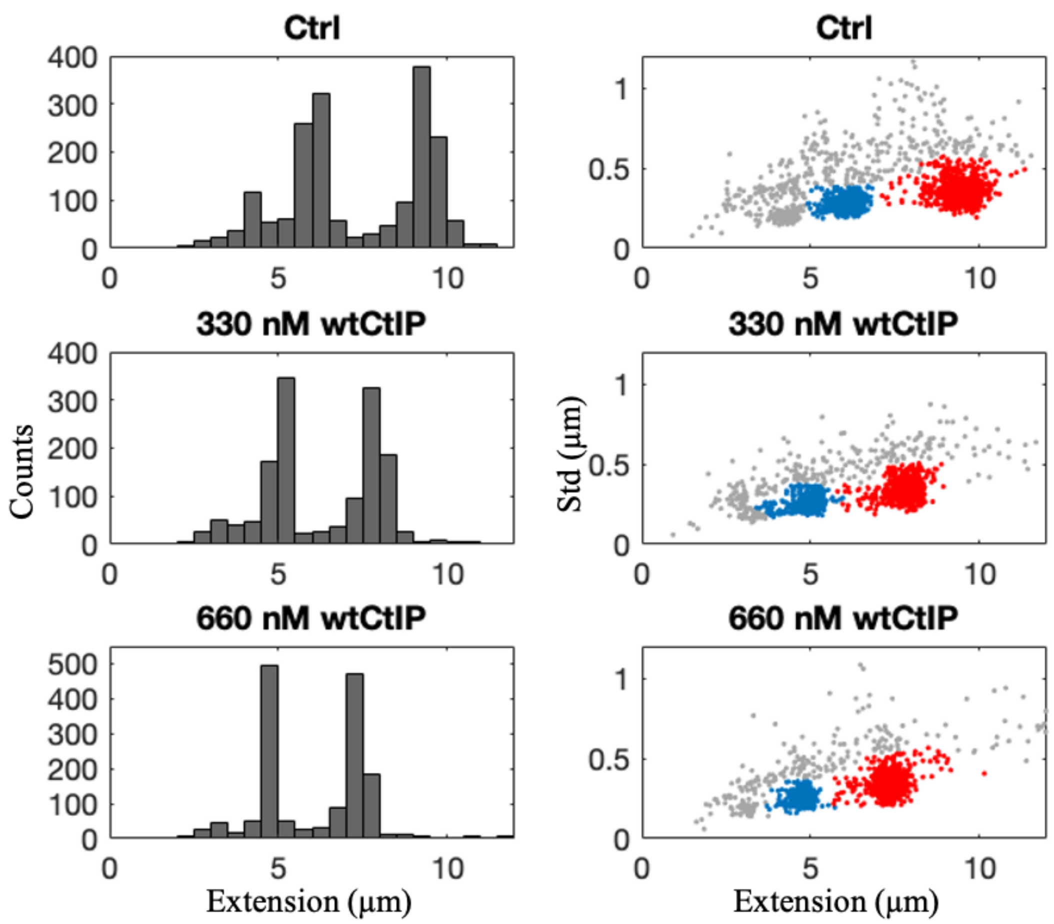

Fig. S14. Histograms of the distribution of DNA-wtCtIP extensions for a plasmid sample (4 μM) at different concentrations of wtCtIP, along with the corresponding scatter plots. The two known plasmids; 62 kbp (blue) and 97 kbp (red) are highlighted in the scatter plot. Other potential plasmids and DNA fragments are colored in grey. Bin-size 0.5 μm.

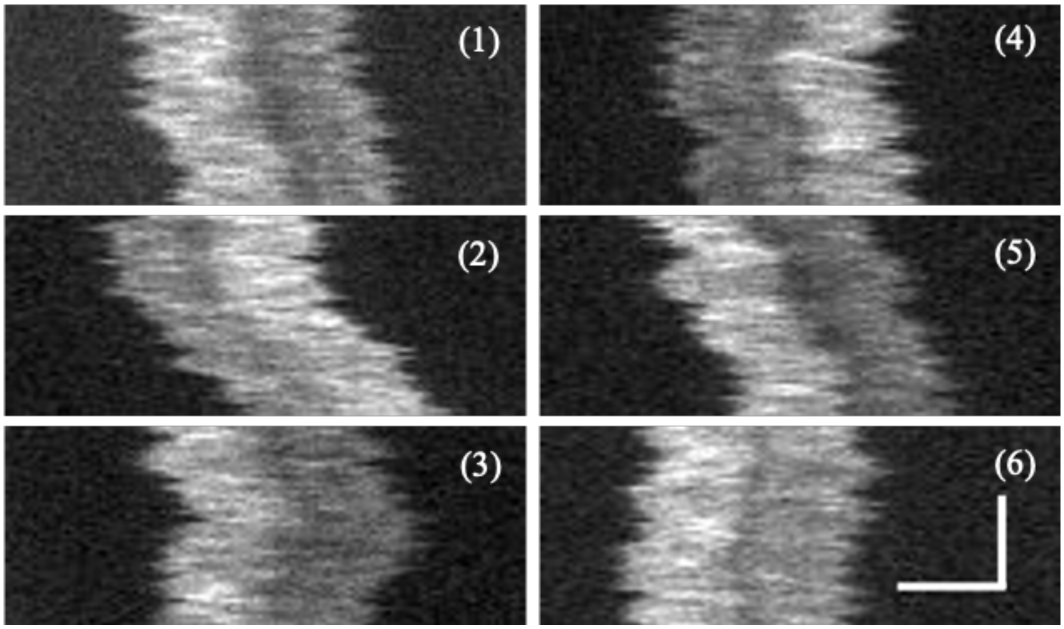

Fig. S15. Kymographs of linear λ-DNA molecules in the presence of wtCtIP at a DNA concentration of 4 μM (bp, equivalent to 500 tetramers per DNA end). No distinct local compactions can be observed along the extension of the molecule. The characteristic signal intensity variations along the extension appears due to sequence dependent heterogenic labelling of YOYO-1, where AT-rich regions are less efficiently labelled compared to GC-rich regions. These intensity variations are masked in circularized λ-DNA molecules due to their double-folded nature. The vertical and horizontal scale-bars correspond to 3 s and 3 μm, respectively.

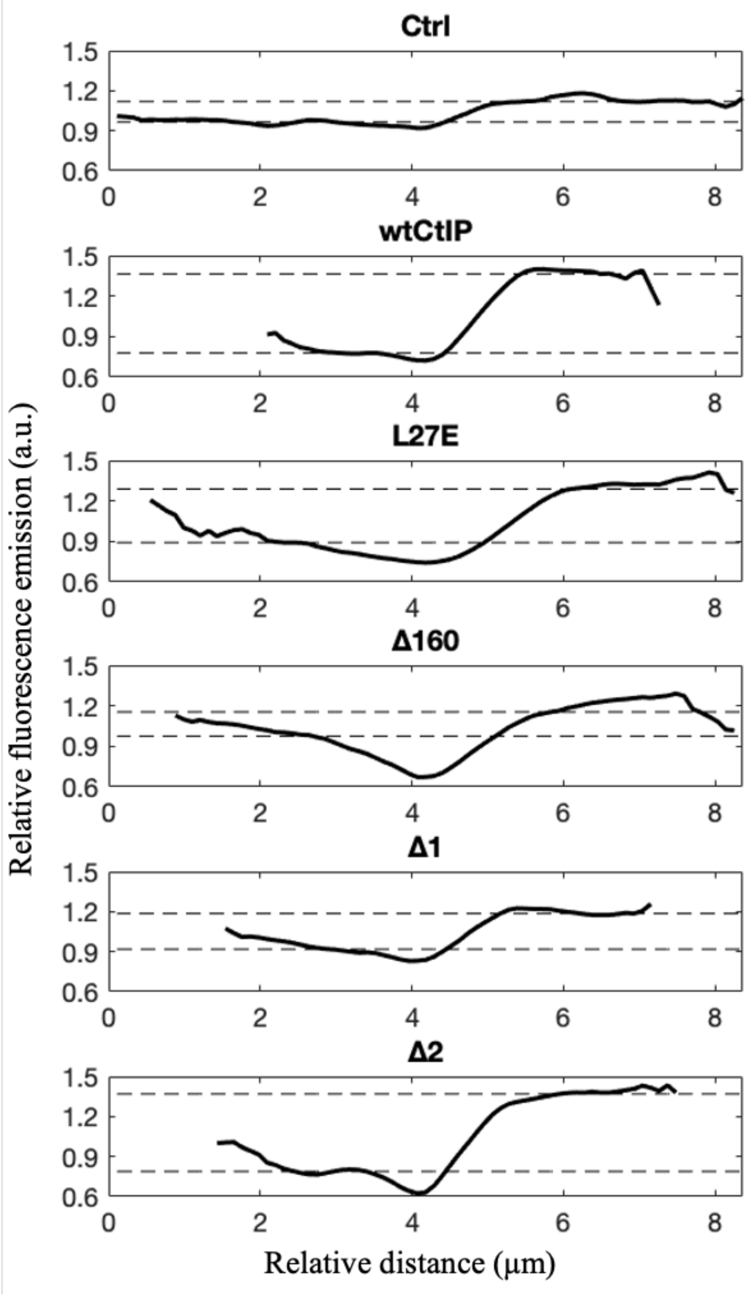

Fig. S16. Relative fluorescence emission profiles along the molecule extension for visually picked kymographs (see Methods section for details) for all CtIP variants. Dashed lines represent the median fluorescence emission values for the AT-rich region (first half of the extension, lower line) and GC-rich region (second half of the extension, upper line). Median values are presented in Tab. S4.

Table S4. Median relative fluorescence emission intensity values for the GC-rich region (first half of the molecule) and AT-rich region (second half of the molecule) for visually picked molecules presented in Fig. S16. The difference in emission intensity reflects the relative amount of YOYO-1 bound to the DNA. All proteins were incubated at the same DNA:protein ratio corresponding to 500 tetramers/1000 dimers/2000 monomers per DNA end (4 μM DNA (bp)).

	**Ctrl λ-DNA**	**wtCtIP 330 nM**	**L27E 330 nM**	**Δ160 330 nM**	**Δ1 330 nM**	**Δ2 330 nM**
Median GC (a.u.)	1.12	1.36	1.29	1.15	1.19	1.37
Median AT (a.u.)	0.97	0.78	0.89	0.98	0.92	0.79
Δ (GC-AT)	0.15	0.59	0.40	0.18	0.27	0.58
N	50	96	36	31	27	163

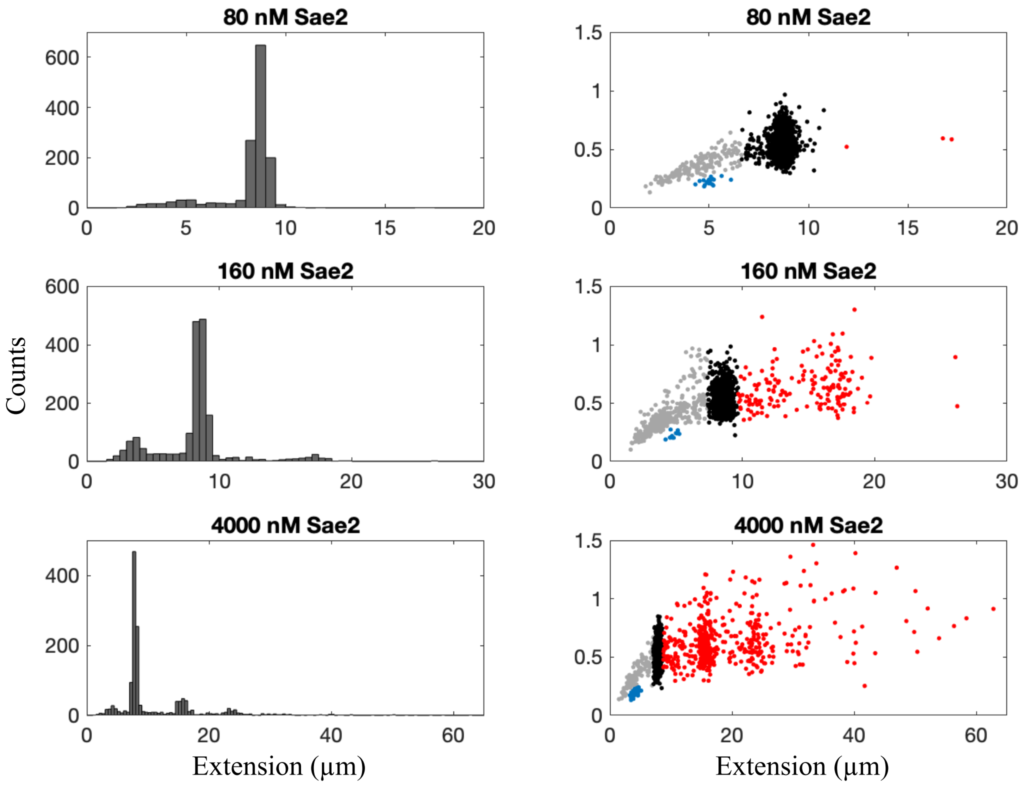

Fig. S17. Size histograms and scatter plots for 4 μM λ-DNA (bp) incubated with Sae2 at different concentrations. Clustering of the datasets was performed to distinguish the circular molecules (blue) from the full-size linear λ-DNA molecules (black), concatemers (red) and fragments (grey). Bin-size 0.5 μM.

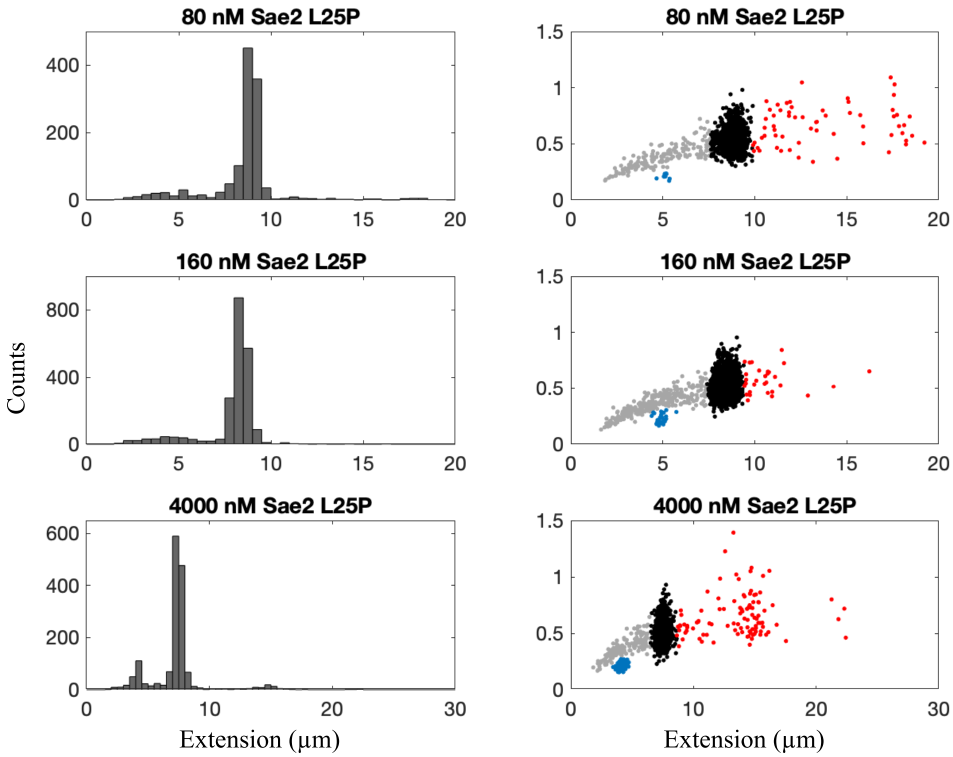

Fig. S18. Size histograms and scatter plots for 4 μM λ-DNA (bp) incubated with $Sae2_{L25P}$ at different concentrations. Clustering of the datasets was performed to distinguish the circular molecules (blue) from the full-size linear λ-DNA molecules (black), concatemers (red) and fragments (grey). Bin-size 0.5 μM.

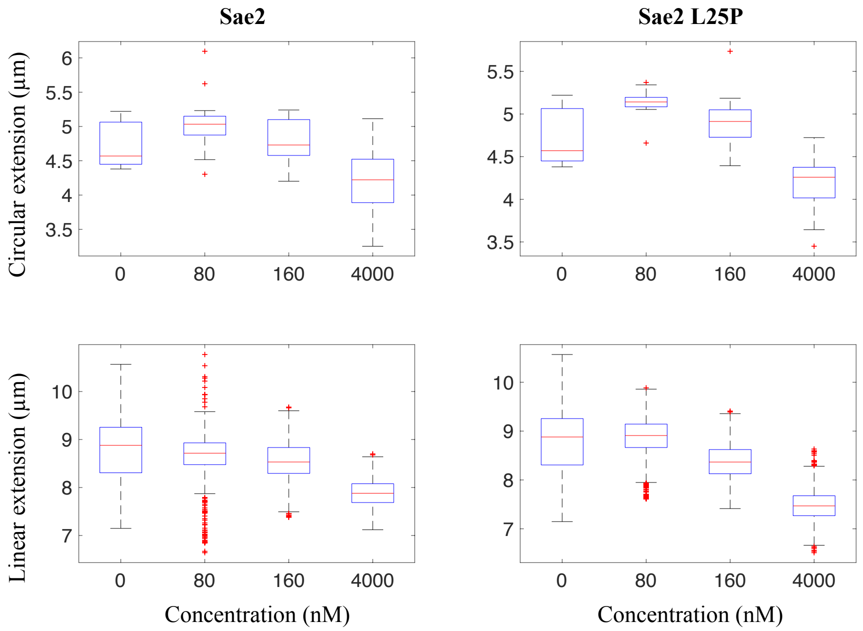

Fig. S19. Boxplot of the distribution of extensions for the circular (top row) and linear (bottom row) fractions of λ-DNA-Sae2 complexes (left column) and λ-DNA-Sae2$_{L25P}$ complexes (right column) at different protein concentrations and a DNA concentration of 4 µM (bp). The blue boxes show the interquartile range (Q2 = 25th percentile, Q3 = 75th percentile) with the median extension (red). Whiskers represent ranges for minimum and maximum and outliers are represented by red crosses. Datapoints deviating by 1.5 times of the interquartile range are considered as outliers.

References

1. Persson, F. & Tegenfeldt, J.O. DNA in nanochannels-directly visualizing genomic information. *Chemical Society Reviews* **39**, 985-999 (2010).
2. Frykholm, K. et al. Fast size-determination of intact bacterial plasmids using nanofluidic channels. *Lab Chip* **15**, 2739-2743 (2015).
3. Nyberg, L.K. et al. Rapid identification of intact bacterial resistance plasmids via optical mapping of single DNA molecules. *Scientific Reports* **6**, 30410 (2016).
4. David, N. & Petr, K. Gwyddion: an open-source software for SPM data analysis. *Open Physics* **10**, 181-188 (2012).
5. Cannavo, E. & Cejka, P. Sae2 promotes dsDNA endonuclease activity within Mre11–Rad50–Xrs2 to resect DNA breaks. *Nature* **514**, 122-125 (2014).
6. Pinto, C., Anand, R. & Cejka, P. in Methods in Enzymology, Vol. 600. (eds. M. Spies & A. Malkova) 67-106 (Academic Press, 2018).
